# ﻿Taxonomy, phylogeny, and biodiversity of Lumbrineridae (Annelida, Polychaeta) from the Central Pacific Clarion-Clipperton Zone

**DOI:** 10.3897/zookeys.1172.100483

**Published:** 2023-07-25

**Authors:** Lenka Neal, Emily Abrahams, Helena Wiklund, Muriel Rabone, Guadalupe Bribiesca-Contreras, Eva C. D. Stewart, Thomas G. Dahlgren, Adrian G. Glover

**Affiliations:** 1 Life Sciences Department, Natural History Museum, London SW7 5BD, UK Natural History Museum London United Kingdom; 2 Department of Marine Sciences, University of Gothenburg, Box 463, 40530 Gothenburg, Sweden University of Gothenburg Gothenburg Sweden; 3 Gothenburg Global Biodiversity Centre, Box 463, 40530 Gothenburg, Sweden Gothenburg Global Biodiversity Centre Gothenburg Sweden; 4 School of Ocean and Earth Sciences, University of Southampton, Southampton, SO14 3ZH, UK University of Southampton Southampton United Kingdom; 5 NORCE Norwegian Research Centre, Bergen, Norway NORCE Norwegian Research Centre Bergen Norway

**Keywords:** CCZ, COI, deep-sea mining, Eunicida, morphology, systematics, phylogeny, 16S, 18S

## Abstract

The DNA taxonomy of six species of the annelid family Lumbrineridae collected from the Clarion-Clipperton Zone (CCZ) in the Central Pacific, an area of potential mining interest for polymetallic nodules, is presented. Lumbrinerids are an ecologically important and understudied annelid family within the deep sea, with many species still undescribed. This study aims to document the taxonomy and biodiversity of the CCZ using specimens collected from the UK-1, OMS, and NORI-D exploration contract areas and Areas of Particular Environmental Interest. Species were identified through a combination of morphological and molecular phylogenetic analysis. We present informal species descriptions associated with voucher specimens, accessible through the Natural History Museum (London) collections, to improve future taxonomic and biodiversity studies of this region. Five taxa in this study had no morphological or genetic matches within the literature and therefore are possibly new to science, but their suboptimal morphological preservation prevented the formalisation of new species. The most abundant taxon Lumbrineridescf.laubieri (NHM_0020) was compared with the holotype of *Lumbrinerideslaubieri* Miura, 1980 from the deep Northeast Atlantic. Currently no reliable morphological characters separating the Pacific and Atlantic specimens have been found and molecular data from the Atlantic specimens was not available.

## ﻿Introduction

The deep sea is a vast and poorly explored habitat that contains both biological and geological novelty. One important geological feature found in the abyssal deep sea are polymetallic nodules containing high grade deposits of metals such as cobalt and nickel (Hein et al. 2013). In the last decade there has been renewed interest in exploration of potential polymetallic nodule mining areas from governments and private investors. The main region of increasing activity is the Clarion-Clipperton Zone (CCZ) in the Central Pacific, a 6 million km^2^ area that lies in the high seas beyond national jurisdiction (ISA 2010). As such, any activities there are regulated by the International Seabed Authority under the United Nations Convention on the Law of the Sea. Essential to the regulatory process is gathering baseline environmental data (Rabone et al. 2023a). In this study, areas of the eastern CCZ surveyed by UK Seabed Resources Ltd (UKSRL), Ocean Mineral Singapore (OMS) and Nauru Ocean Resources Inc (NORI-D) were targeted. Additional material was also collected from a region excluded from mining contracts, Area of Particular Environmental Interest number 6 ‘APEI-6’.

The CCZ abyssal seafloor is characterised by soft sediments mixed with hard-substrate polymetallic nodules. The annelids dominate the macrofaunal size range of benthos contributing just over half the fauna by abundance and species richness, with many species undescribed (Rabone et al. 2023b). Taxonomic knowledge is key to future environmental risk assessments (Glover et al. 2018) and previous publications from the areas targeted in this study have already yielded 54 annelid species from 267 specimen records, of which 18 species were formalised as new (Wiklund et al. 2019; Drennan et al. 2021; Neal et al. 2022a, b). Across the CCZ as a whole, a total of 52 polychaete species and four genera new to science have now been described (Blake 2016, 2017, 2019, 2020; Paterson et al. 2016; Bonifacio and Menot 2019; Wiklund et al. 2019; Maciolek 2020; Drennan et al. 2021; Neal et al. 2022a, b); compiled into the first checklist for the region (Rabone et al. 2023b). With this in mind, we present a taxonomic study of Lumbrineridae Schmarda, 1861, an abundant, yet often overlooked, annelid family.

Currently, Lumbrineridae are represented by 279 species, and 19 genera (Zanol et al. 2021). Historically, many species within Lumbrineridae have been incorrectly hypothesised to have a “cosmopolitan” geographic and bathymetric distribution as common for other annelid taxa. For example, use of the same keys to describe species from geographically distinct regions has led to the incorrect use of names for local species (Carrera-Parra 2001). This has created a problematic taxonomic history, with many genera poorly described. There are very few phylogenetic studies of Lumbrineridae, and many genera require revisions based on the maxillary apparatus and molecular data (Zanol et al. 2021). Carrera-Parra (2006a) provided phylogenetic reconstruction of lumbrinerid genera using parsimony analyses of 38 morphological characters. Remarkably, the first molecular phylogenetic study on Lumbrineridae was only carried out as recently as 2022 (Borisova and Budaeva 2022), albeit based on limited taxon sampling. They recovered genera *Abyssoninoe* Orensanz, 1990; *Augeneria* Monro, 1930; *Gallardoneris* Carrera-Parra, 2006a; *Lumbrineriopsis* Orensanz, 1973 and *Ninoe* Kinberg, 1865 as monophyletic, while indicating polyphyly of some genera, including *Lumbrineris*, the type genus of the family.

Morphologically, lumbrinerids are elongated cylindrical worms with complex jaws (Oug 2011; Oug et al. 2022). The family has a history of being poorly described since many external characters are reduced (Carrera-Parra 2004). Today they are primarily characterised by their maxillary apparatus, which is a key taxonomic feature for the family (Oug 2011; Zanol et al. 2021; Oug et al. 2022). Previously most genera were defined by the presence or absence of chaetal types. Some genera have been revised and redefined in recent years to include the maxillary apparatus. For example, Carrera-Parra (2004) redefined the genus *Lumbricalus* using this approach. To date, five genera of lumbrinerids have undergone systematic revision (or partial revision): *Kuwaita* Mohammad, 1973 (Carrera-Parra and Orensanz 2002), *Lumbrineris* de Blainville, 1828 (Carrera-Parra 2006b), *Lumbrinerides* Orensanz, 1973 (Perkins 1979; Miura 1980, 2017), *Lumbrineriopsis* Orensanz, 1973 (Miura 1980) and *Lumbricalus* Frame, 1992 (Carrera-Parra 2004). The remaining genera await further revision and phylogenetic analysis based on maxillary apparatus and genetic data. Aside from the maxillary apparatus and chaetae type and shape, other key taxonomic features of Lumbrineridae include colour of the aciculae, the shape of the parapodial lobes, presence or absence of branchiae, and presence or absence of antennae and eyes (Oug 2011, 2012; Oug et al. 2022). Some genera, such as *Lumbrinerides* and *Lumbrineriopsis* have distinctive elongated prostomiums, though prostomium shape is not often used as a diagnostic character (Hilbig 1995). Additionally, the presence of antenna, palps and eyes distinguishes the genera *Lysarete* and *Kuwaita* from the rest of the family (Hilbig 1995). The recent molecular phylogenetic work of Borisova and Budaeva (2022) suggests that morphological characters traditionally used in lumbrinerid systematics (e.g., presence of connecting plates, four pairs of maxillae, bidentate simple hooded hooks, colourless maxillae IV, and multidentate maxillae IV) have probably evolved independently within Lumbrineridae several times.

In terms of their ecology, Lumbrineridae are generally considered carnivores, deposit-feeders, or scavengers (Barnes et al. 1979; Oug et al. 2022). The musculature and structure of the jaw indicates that food is gathered by both sucking and grasping manoeuvres (Hilbig 1995). They are mostly found in soft sedimentary habitats, burrowing through the sediment, and constructing mucus-lined tubes (Hilbig 1995; Oug 2011; Zanol et al. 2021; Oug et al. 2022). Their reproduction and development are understudied, but of the species where reproductive mode is known they are all gonochoric and without sexual dimorphism (Zanol et al. 2021; Oug et al. 2022).

Lumbrinerids are abundant globally and numerous within CCZ samples taken over the last 30 years, and they are a potential useful indicator taxon due to their recognisability and high abundance. For example, some species within Lumbrineridae may be able to act as environmental indicators of disturbance (Borowski and Thiel 1998; Giangrande et al. 2005) or models for estimates of population connectivity (Stewart et al. 2023). They are also bioturbators and important prey (Zanol et al. 2021). This study aimed to recover the phylogeny of lumbrinerids collected across the CCZ, and present publicly available DNA sequences, images, and taxonomic descriptions of species for future use, which in turn allows us to understand better their contribution to abyssal diversity and ecology within the CCZ.

## ﻿Materials and methods

### ﻿Fieldwork

**Figure 1.**
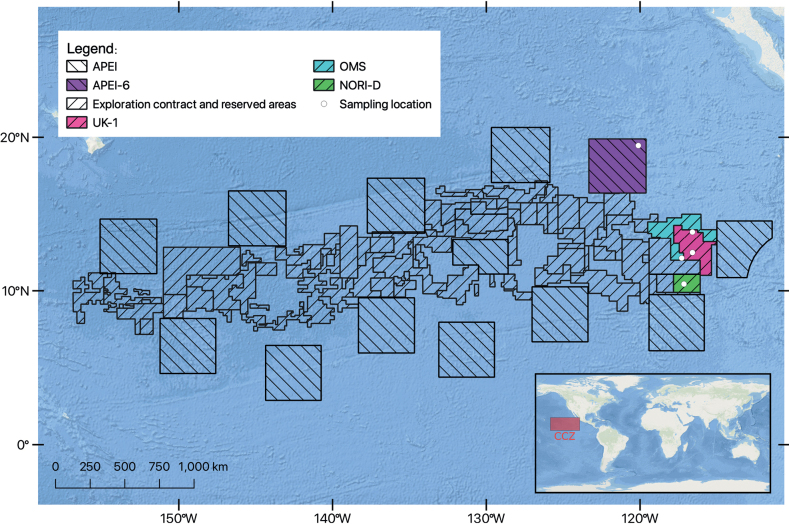
Map of CCZ of exploration areas and Areas of Particular Environmental Interest (APEI) with targeted areas (highlighted in colours; see legend) where samples for this study were collected.

The first UKSR ABYSSLINE cruise (AB01) took place in October 2013 onboard the RV ‘Melville’ and targeted the UK-1 exploration contract area (Fig. 1). The second cruise (AB02) took place in February-March 2015 onboard RV ‘Thomas G. Thompson’ and sampled a wider area (Fig. 1), including: the UK-1 (depth ~ 4200 m) and OMS (depth ~ 4200 m) exploration contract areas and APEI-6 (depth ~ 4050 m), an area exempted from mining activities (Wedding et al. 2013). The Resource Cruise 01 (RC01) took place aboard the marine vessel M/V ‘Pacific Constructor’ between February and March 2020 and targeted exploration contract areas UK-1 and OMS (Fig. 1). Nauru Ocean Resources Inc (NORI) Campaign 05a (DG05a) cruise took place between October and November 2020 and the 05d (DG05d) cruise took place between April and June 2021; both expeditions were onboard ‘Maersk Launcher’ to the NORI-D exploration contract area (depth ~ 4300 m) (Fig. 1).

For a comprehensive description of the methodological pipeline, see Glover et al. (2016). Briefly, specimens were collected using box corer and Brenke epibenthic sledge (EBS) (Brenke 2005). Geographic data from sampling activities were recorded on a central GIS database. Live-sorting of specimen samples was carried out onboard all four vessels in a ‘cold-chain’ pipeline, with material maintained in chilled (2–4 °C), filtered seawater. Specimens were preliminarily identified and imaged live using stereo microscopes with attached digital cameras (Glover et al. 2016). Specimens were then stored in individual appropriately labelled microtube vials filled with aqueous solution of 80% non-denatured ethanol and entered into a local database.

### ﻿Laboratory work

Laboratory work was carried out using facilities at the Natural History Museum, London and University of Gothenburg, Sweden. Sixty preserved specimens were examined using stereo and compound microscopes. Five specimens lacked heads and were identified by molecular data only (see below). Fifty-five specimens were identified to morphospecies, and the best-preserved examples (voucher specimens) were then used to provide informal descriptions with key morphological features photographed with a digital camera. Shirlastain A was used during the morphological examination on some specimens to better observe certain characters. For some species, we dissolved out the jaws from the specimens to capture a clearer image of their structure. The anterior end was decapitated and transferred into a small amount of porcine Trypsin solution (1 ml borax, 1 ml distilled water, 0.5 mg of porcine Trypsin). Specimens were left between 15–45 min in the solution depending on their size before transfer onto a droplet of distilled water on a microscope slide and held in place with a cover slip. The jaws could then be viewed and photographed in detail using a compound microscope. Jaws were dissolved from eight specimens, preserved on a permanent slide a given NHMUK registration number (Table 1). Figures were assembled using Adobe Photoshop CS6 software.

**Table 1.** List of taxa presented in this paper - taxonConceptID (a species-level identification based on combined DNA and morphological evidence), cruise record number, GUID (Global Unique Identifier, linking to data record at http://data.nhm.ac.uk), NHMUK registration number, NHMUK Molecular Collection facility (MCf) sample ID number (NHMUK MCf no.), and NCBI GenBank accession number (GenBank Acc. no.) for successfully sequenced genetic markers. GenBank numbers for phylogenetic analysis data downloaded from GenBank are presented in Suppl. material 1.

TaxonConceptID	NHM no.	GUID	NHM Reg. no.	COI Acc. no.	18S Acc. no.	16S Acc. no.	NHMMCf no.
Lumbrineridae sp. NHM_2146	NHM_2146	2c8356c2-f64f-4a40-ac0b-292cfc5247b6	ANEA 2022.855	OQ857795	OQ865007	OQ865035	109405345
Lumbrineridae sp. NHM_1485	NHM_1485	f44e9778-31b9-4204-8b41-f2229302bfd3	ANEA 2022.852	OQ857790	OQ865005	OQ865027	109405344
Lumbrineridae sp. NHM_1485	NHM_1516	18dad4b1-fb07-49ef-a56a-168ced070d7f	ANEA 2022.853	OQ857791		OQ865028	109405438
Lumbrineridae sp. NHM_1485	NHM_1843	8250bf25-7734-4db0-b18c-6ad49e5cc9f6	ANEA 2022.854	OQ857793		OQ865030	109405414
*Augeneria* sp. NHM_4590	NHM_0205	2af3e568-87be-4158-9b8e-f5b1c3cc5468	ANEA 2022.832			OQ865012	118302149
*Augeneria* sp. NHM_4590	NHM_1008	9338cf46-ea85-43d8-9593-1d2395acb4ca	ANEA 2022.833	OQ857787		OQ865024	109405368
*Augeneria* sp. NHM_4590	NHM_2389	5cd29524-9ddf-43a0-8618-5ec8f6dc0bf8	ANEA 2022.834	OQ857797		OQ865039	109405370
*Augeneria* sp. NHM_4590	NHM_3886	10ae7a30-345f-40b0-9fb6-ec84eb4d91d5	ANEA 2022.835	OQ857800		OQ865044	109405412
*Augeneria* sp. NHM_4590	NHM_2249	d2bc88ee-e882-4f6d-aa4c-93bcdb429611	ANEA 2022.836			OQ865036	109405413
*Augeneria* sp. NHM_4590	NHM_4590	4ed5a4c7-a44a-4cce-8e0b-4a5036fd5b5c	ANEA 2022.837	OQ857802	OQ865008	OQ865046	109405388
*Augeneria* sp. NHM_4590	NHM_2588	22fbc1a3-b2bd-40c2-bf7f-d3dcd6b85ea4	ANEA 2022.838				109405346
*Augeneria* sp. NHM_4590	NHM_2976	efc8baac-defe-468f-a40d-5b0c2e160621	ANEA 2022.839				
*Augeneria* sp. NHM_4590	NHM_4738_ECDS4	fc4ac1a4-1a78-475f-a9bc-36740d2e1bd9	ANEA 2022.840	OQ857806			109405435
*Augeneria* sp. NHM_4590	NHM_0209	1b024172-3aba-4404-9cd5-6f9cc86d69c0		OQ857783		OQ865013	118302150
*Augeneria* sp. NHM_4590	NHM_0609	98e583f4-665d-4197-8349-c8f6147454b8				OQ865016	118302153
*Augeneria* sp. NHM_4590	NHM_0686	1b1830d0-ec8f-4503-86ca-72efba0a4772	ANEA 2022.841			OQ865017	118302154
*Augeneria* sp. NHM_4590	NHM_0782	10adaf27-6b76-4258-8530-5cb8ef631c44	ANEA 2022.842			OQ865020	109405416
*Augeneria* sp. NHM_4590	NHM_0788	87c4f766-d47b-4bf4-85e3-b5e234cdf241	ANEA 2022.843			OQ865021	109405415
*Augeneria* sp. NHM_4590	NHM_1872	621a4712-aea8-42c6-8ad9-ee673f0d06c6	ANEA 2022.844			OQ865031	109405393
*Augeneria* sp. NHM_4590	NHM_1878	cd9d9fd6-547d-4b98-b111-d47e647333bf	ANEA 2022.845			OQ865032	109405390
*Augeneria* sp. NHM_4590	NHM_1948K	8e37077f-b5ff-4190-a75e-23dd07314798	ANEA 2022.846			OQ865034	109405366
*Augeneria* sp. NHM_0851	NHM_0420	bde33da0-8f07-470a-b63a-979326313974				OQ865015	118302152
*Augeneria* sp. NHM_0851	NHM_0761	52a58501-7bfc-4dcc-af95-0509a6463600	ANEA 2022.847			OQ865019	109405439
*Augeneria* sp. NHM_0851	NHM_0851	71820a17-178d-4fcd-b493-8fac5f8b45cb	ANEA 2022.848	OQ857785	OQ865004	OQ865022	109405392
*Augeneria* sp. NHM_0851	NHM_2441	a0b38651-82c1-4410-a352-33da931260ad	ANEA 2022.849			OQ865040	109405365
*Augeneria* sp. NHM_0851	NHM_0737	a9119f6d-0787-40a4-87cd-08cc80d8b35b	ANEA 2022.850			OQ865018	118302155
Lumbrineridescf.laubieri	NHM_0020	cfc84885-578e-4b87-a14f-e8fc1cf2a5a0	ANEA 2022.801	OQ857779	OQ865002	OQ865009	118300518
Lumbrineridescf.laubieri	NHM_0028	0adcf12b-4027-4893-98a2-588d60e352a3	ANEA 2022.802	OQ857780			118302145
Lumbrineridescf.laubieri	NHM_1146	4849b790-53a1-45f8-a7e0-3f60062642cd	ANEA 2022.803	OQ857788		OQ865025	118302146
Lumbrineridescf.laubieri	NHM_3492	e6b00cf4-5c01-453d-a1ff-5c7dc0783960	ANEA 2022.804			OQ865042	109405436
Lumbrineridescf.laubieri	NHM_2245	024a6d28-6775-4d01-823b-3b0a03fcd727	ANEA 2022.805				109405418
Lumbrineridescf.laubieri	NHM_4738_ECDS5	2806a25b-e00e-4845-9b6a-10ca95b3fd6a	ANEA 2022.806	OQ857807			109405420
Lumbrineridescf.laubieri	NHM_4738_ECDS3	7d7e9048-38fa-4503-b256-6e6ac3c23687	ANEA 2022.807	OQ857805			109405347
Lumbrineridescf.laubieri	NHM_4738_ECDS1	c78086ef-af8a-4b1c-b9db-e61df25585f4	ANEA 2022.808	OQ857803			109405371
Lumbrineridescf.laubieri	NHM_4743_ECDS1	d42c9eb3-2052-4e90-9e00-61e20291d674	ANEA 2022.809	OQ857808			109405411
Lumbrineridescf.laubieri	NHM_8798_HW02	568f9a43-2c36-49be-8829-55edb69ba271	ANEA 2022.810	OQ857810			109405348
Lumbrineridescf.laubieri	NHM_8777_HW01	18c5a2d0-2bd6-48e2-bd02-9fe7c03ae7a4	ANEA 2022.811			OQ865048	109405372
Lumbrineridescf.laubieri	NHM_8898_LN01	af52e52a-9c50-432e-a88c-1f03cb6f981c	ANEA 2022.812				
Lumbrineridescf.laubieri	NHM_8898_LN02	123e1f28-a76e-4929-9c58-7ffd80db14a2	ANEA 2022.813				
Lumbrineridescf.laubieri	NHM_8898_LN03	2c462f72-683e-4fb3-bef9-8a8fe10b98f3	ANEA 2022.814				
Lumbrineridescf.laubieri	NHM_8810	75d2356a-cfd2-4ea8-b6d4-7d69ee27cb1c	ANEA 2022.815				
Lumbrineridescf.laubieri	NHM_8855	2250d689-4b91-4998-8776-05db9d437c89	ANEA 2022.816				
Lumbrineridescf.laubieri	NHM_8874	4d437d57-72b5-4f7a-84b7-87b13b6c422d	ANEA 2022.817				
*Lumbrineris* sp. NHM_1741	NHM_0125	067a1dee-adcb-4c7f-8445-b68176b5c41b		OQ857781		OQ865010	118302147
*Lumbrineris* sp. NHM_1741	NHM_0229	9474abbe-8d3a-4db8-9897-ff206977918f	ANEA 2022.818	OQ857784	OQ865003	OQ865014	118302151
*Lumbrineris* sp. NHM_1741	NHM_1741	86010404-eae6-4c58-943a-1761c81fa201	ANEA 2022.819	OQ857792	OQ865006	OQ865029	109405417
*Lumbrineris* sp. NHM_1741	NHM_0972	c570a340-f2e6-405d-b0b8-f335c9056fbd	ANEA 2022.820	OQ857786		OQ865023	109405391
*Lumbrineris* sp. NHM_1741	NHM_2318	83e28d46-6d21-4fe6-a9a6-2c7360cfbaa4	ANEA 2022.821	OQ857796		OQ865037	109405394
*Lumbrineris* sp. NHM_1741	NHM_2374	f1cda422-c3a0-47cb-b63a-40a1d92232d7	ANEA 2022.822			OQ865038	109405389
*Lumbrineris* sp. NHM_1741	NHM_4237	be32a0a9-56ef-41fc-ae65-cf939128372b	ANEA 2022.823	OQ857801		OQ865045	109405395
*Lumbrineris* sp. NHM_1741	NHM_3591	23c92bc4-3099-45b9-bc63-455e094fd5c7	ANEA 2022.824	OQ857799		OQ865043	109405419
*Lumbrineris* sp. NHM_1741	NHM_4738_ECDS2	cd4b907b-383e-4a71-8ebb-573a8745a8bc	ANEA 2022.825	OQ857804			109405364
*Lumbrineris* sp. NHM_1741	NHM_8796_HW01	30460632-eb9b-4655-81c2-c1ce3b2cebdd	ANEA 2022.826			OQ865049	109405363
*Lumbrineris* sp. NHM_1741	NHM_7057_HW01	50895550-0014-46d9-b0ba-76b9e392281b	ANEA 2022.827	OQ857809			109405396
*Lumbrineris* sp. NHM_1741	NHM_3133	0f6f9455-d82a-4afa-9a12-5f6771e66763	ANEA 2022.828	OQ857798		OQ865041	109405437
*Lumbrineris* sp. NHM_1741	NHM_1896	acf2aa8f-3ca5-4728-ab42-782576ab57fd	ANEA 2022.829	OQ857794		OQ865033	109405369
*Lumbrineris* sp. NHM_1741	NHM_1308	2d378a92-05c4-417a-9cc7-1c392baf0db7	ANEA 2022.830	OQ857789		OQ865026	109405367
*Lumbrineris* sp. NHM_1741	NHM_0129	3c704b88-d8ae-42cb-aeae-73e7a20a70b7		OQ857782		OQ865011	118302148
*Lumbrineris* sp. NHM_1741	NHM_7249_HW01	73e121b5-97fb-4d6e-905f-4387236b9cf6	ANEA 2022.831			OQ865047	109405387
*Lumbrineri*s sp. NHM_1741	NHM_8899	462dea9c-52cb-48bb-8e60-43c5f4f5d472	ANEA 2022.851	OQ857811			109405434

### ﻿Molecular laboratory work

Molecular data were obtained from 53 specimens and used to place species covered in this study within Lumbrineridae phylogenetic relationships. Extraction of DNA was done with DNeasy Blood and Tissue Kit (Qiagen) using a Hamilton Microlab STAR Robotic Workstation, or with QuickExtractTM DNA extraction solution (Lucigen), following manufacturer guidelines, and adapted for a digestion time of 40 minutes. Approximately 1800 bp of 18S were amplified using the primers 18SA 5’-AYCTGGTTGATCCTGCCAGT-3’ (Medlin et al. 1988) and 18SB 5’-ACCTTGTTACGACTTTTACTTCCTC-3’ (Nygren and Sundberg 2003). Approximately 450 bp of 16S were amplified with the primers ann16Sf 5’-GCGGTATCCTGACCGTRCWAAGGTA-3’ (Sjölin et al. 2005) and 16SbrH 5’-CCGGTCTGAACTCAGATCACGT-3’ (Palumbi 1996), and ~ 650 bp of cytochrome c oxidase I (COI) were amplified using LCO1490 5’-GGTCAACAAATCATAAAGATATTGG-3’ (Folmer et al. 1994) and COI-E 5’-TATACTTCTGGGTGTCCGAAGAATCA-3’ (Bely and Wray 2004). PCR mixtures contained 1 µl of each primer (10 µM), 2 µl template DNA and 21 µl of Red Taq DNA Polymerase 1.1X MasterMix (VWR) in a mixture of total 25 µl. The PCR amplification profile for all gene fragments consisted of initial denaturation at 95 °C for 5 min, 35 cycles of denaturation at 94 °C for 45 s, annealing at 55 °C for 45 s, extension at 72 °C for 2 min, and a final extension at 72 °C for 10 min. PCR products were purified using Millipore Multiscreen 96-well PCR Purification System, and sequencing was performed on an ABI 3730XL DNA Analyser (Applied Biosystems) at The Natural History Museum Sequencing Facility, using the same primers as in the PCR reactions plus two internal primers for 18S, 620F 5’-TAAAGYTGYTGCAGTTAAA-3’ (Nygren and Sundberg 2003) and 1324R 5’-CGGCCATGCACCACC-3’ (Cohen et al. 1998). Overlapping sequence fragments were merged into consensus sequences using Geneious (Kearse et al. 2012) and aligned using MAFFT (Katoh et al. 2002) for 18S and 16S, and MUSCLE (Edgar 2004) for COI, both programs used as plugins in Geneious, with default settings.

Molecular data were used to place species covered in this study within the lumbrinerid phylogenetic relationships. Sequences added from GenBank are listed in Suppl. material 1. with taxon names and sequence accession numbers. Representatives from the annelid families Eunicidae, Onuphidae, and Oenonidae were used as outgroup. The program jModelTest (Posada 2008) was used to assess the best model for each partition with BIC, which suggested GTR+I+G as the best model for all genes. The data was partitioned into three genes (18S, 16S and COI), and the evolutionary model mentioned above was applied to each partition. The parameters used for the partitions were unlinked. Bayesian phylogenetic analyses (BAs) were conducted with MrBayes v. 3.2.6 (Ronquist et al. 2012). Analyses were run three times for 10,000,000 generations. Of these, the first 2,500,000 generations were discarded as burn-in. The tree files were interpreted with FigTree v. 1.4.4 (available from http://tree.bio.ed.ac.uk/software/figtree/).

### ﻿Taxonomic assignments

We use a conservative approach to species delimitation where morphological data is missing or insufficient, keeping the lowest taxonomic level e.g., genera. We use a phylogenetic species concept, sensu Donoghue 1985, where species are defined by DNA-based phylogenetic analysis and distinctive monophyletic groups are recognised as species.

Species are named informally with the NHM voucher specimen assigned to represent that species. For example, the name *Lumbrineris* sp. NHM_1741 is used to represent all specimens that are the same species as specimen NHM_1741. For species where we lack genetic data, where the morphological data is inconclusive, or where data from GenBank cannot be used to compare to our specimens, we use the open nomenclature term “cf.” to indicate uncertainty. All voucher specimens and DNA extractions were deposited at the Natural History Museum (**NHM**) London (Table 1).

### ﻿Data handling

The field and laboratory work led to a series of databases and sample sets that were integrated into a ‘data-management pipeline’. This included the transfer and management of data and samples between a central collections database, a molecular collections database and external repositories (GenBank, WoRMS, OBIS, GBIF, GGBN, ZooBank) through DarwinCore archives (Suppl. material 2). As this study examines specimens from different cruise programmes, utilising different coordinate systems during site data collection, the geographic coordinates are standardised to decimal degrees in the material examined section, but verbatim coordinates (including decimal minute and UTM) are included in the DarwinCore archive (Suppl. material 2). This provides a robust data framework to support DNA taxonomy, in which openly available data and voucher material are key to quality data standards. A further elaboration of the data pipeline is published in Glover et al. (2016).

## ﻿Results

### ﻿Systematics

**Lumbrineridae Schmarda, 1861**

#### ﻿ Lumbrineridae

Taxon classificationAnimaliaEunicidaLumbrineridae

sp. NHM_2146

32BA5656-2171-5446-A03F-2A1DB02B1E48

Fig. 2A–D

##### Material examined.

NHM_2146, NHM ANEA 2022.855, coll. 20 Mar. 2015, AB02, APEI6, EBS, 19.46457, -120.02542, 4026 m, https://data.nhm.ac.uk/object/2c8356c2-f64f-4a40-ac0b-292cfc5247b6.

##### Description.

**Figure 2.**
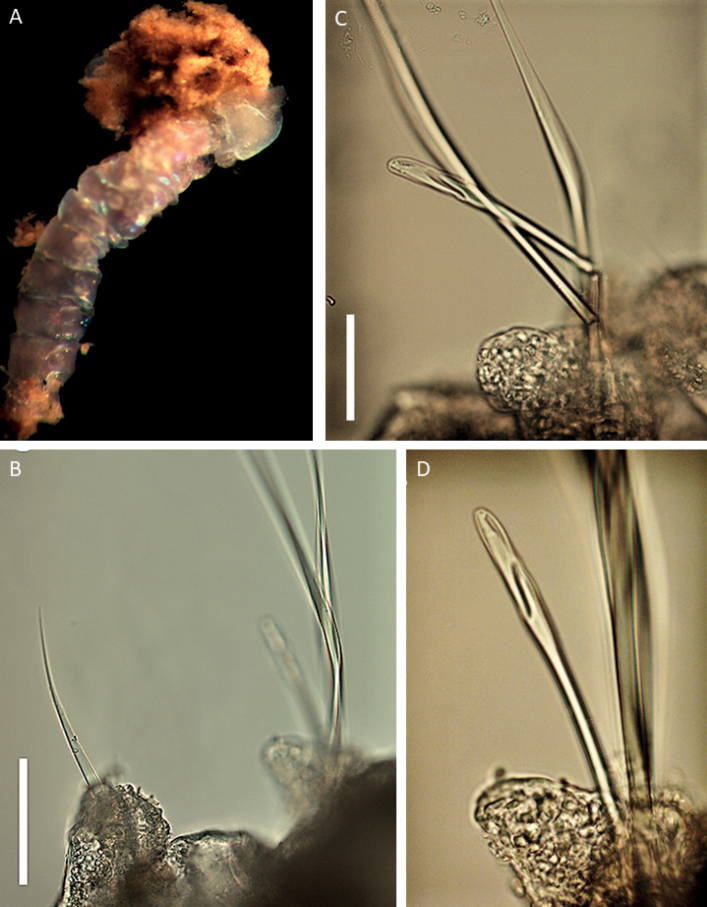
Lumbrineridae sp. NHM_2146 **A** live image of body fragment of specimen NHM_2146 **B** winged limbate capillary chaetae **C** pseudo-compound multidentate hooded hook and winged capillaries **D** pseudo-compound multidentate hooded hook. Scale bars: 100 µm (**B**); 50 µm (**C**).

This species is represented by a single sub-optimally preserved body fragment, 1.8 mm long and 0.4 mm wide for ~ 7 discernible chaetigers (Fig. 2A). Parapodia indistinct, globular parapodial lobe visible on some parapodia (Fig. 2B, C). Aciculae yellow. Chaetae characterised by winged limbate chaetae (Fig. 2C), pseudo-compound multidentate hooded hooks (Fig. 2C, D). No simple hooks visible on specimen, although posterior end is absent, and many chaetae are broken.

##### Genetic data.

**Figure 3.**
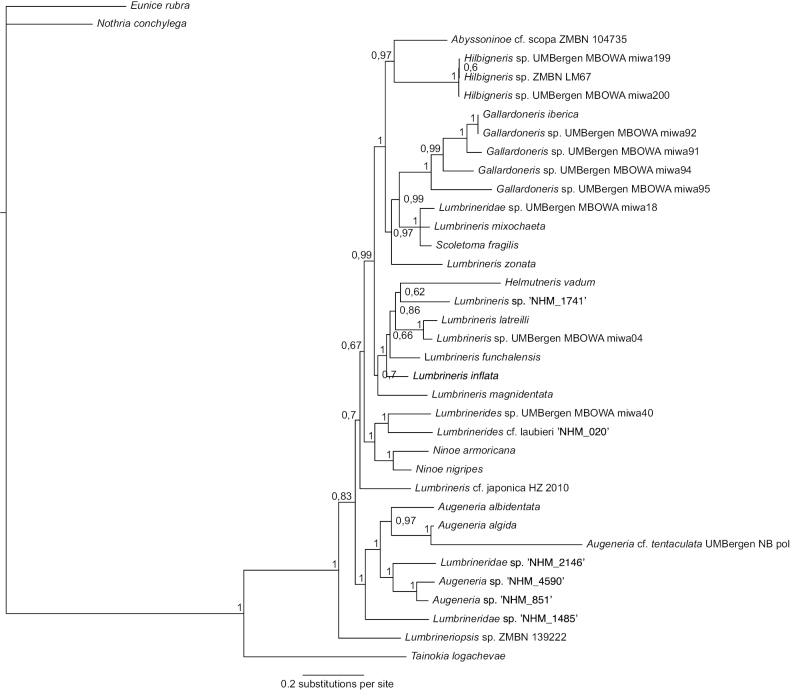
Majority rule consensus tree from the Bayesian analyses using combined datasets for COI, 16S and 18S genes, with 36 terminal taxa of which *Tainokialogachevae* (Oenonidae), *Eunicerubra* (Eunicidae) and *Nothriaconchylega* (Onuphidae) were used as outgroup. Posterior probability values are marked on nodes.

This species falls in a strongly supported clade containing *Augeneria* species, suggesting it may belong to this genus (Fig. 3). There are no matches for this species on GenBank.

##### Remarks.

Due to the suboptimal quality of the single available specimen, we cannot identify this taxon beyond family level using morphology. Prostomium and jaws cannot be observed. Chaetae are characterised by winged limbate chaetae and hooks that appears to be pseudo-compound and multidentate. No simple hooks visible on specimen, although posterior end is absent, and many chaetae are broken. Molecular data suggest this species may belong to genus *Augeneria* (Fig. 3), but given the lack of morphological data, we cautiously assign this specimen to morphospecies Lumbrineridae sp. NHM_2146.

##### Distribution.

Central Pacific Ocean, Eastern CCZ, in the Area of Particular Environmental Interest, ‘APEI-6’ only (Fig. 1).

#### ﻿ Lumbrineridae

Taxon classificationAnimaliaEunicidaLumbrineridae

sp. NHM_1485

15D9C81C-86E5-5C7B-8C58-725F569430EC

Figs 4A–E , 5A–F

##### Material examined.

NHM_1485, NHM ANEA 2022.852, coll. 4 Mar. 2015, AB02, UK-1, Box core, 12.495, -116.65018, 4260 m, https://data.nhm.ac.uk/object/f44e9778-31b9-4204-8b41-f2229302bfd3; NHM_1516, NHM ANEA 2022.853, coll. 5 Mar. 2015, AB02, UK-1, EBS, 12.51317, -116.49133, 4252 m, https://data.nhm.ac.uk/object/18dad4b1-fb07-49ef-a56a-168ced070d7f; NHM_1843, NHM ANEA 2022.854, coll. 13 Mar. 2015, AB02, OMS, Megacore, 12.05465, -117.25158, 4096 m, https://data.nhm.ac.uk/object/8250bf25-7734-4db0-b18c-6ad49e5cc9f6.

##### Description.

**Figure 4.**
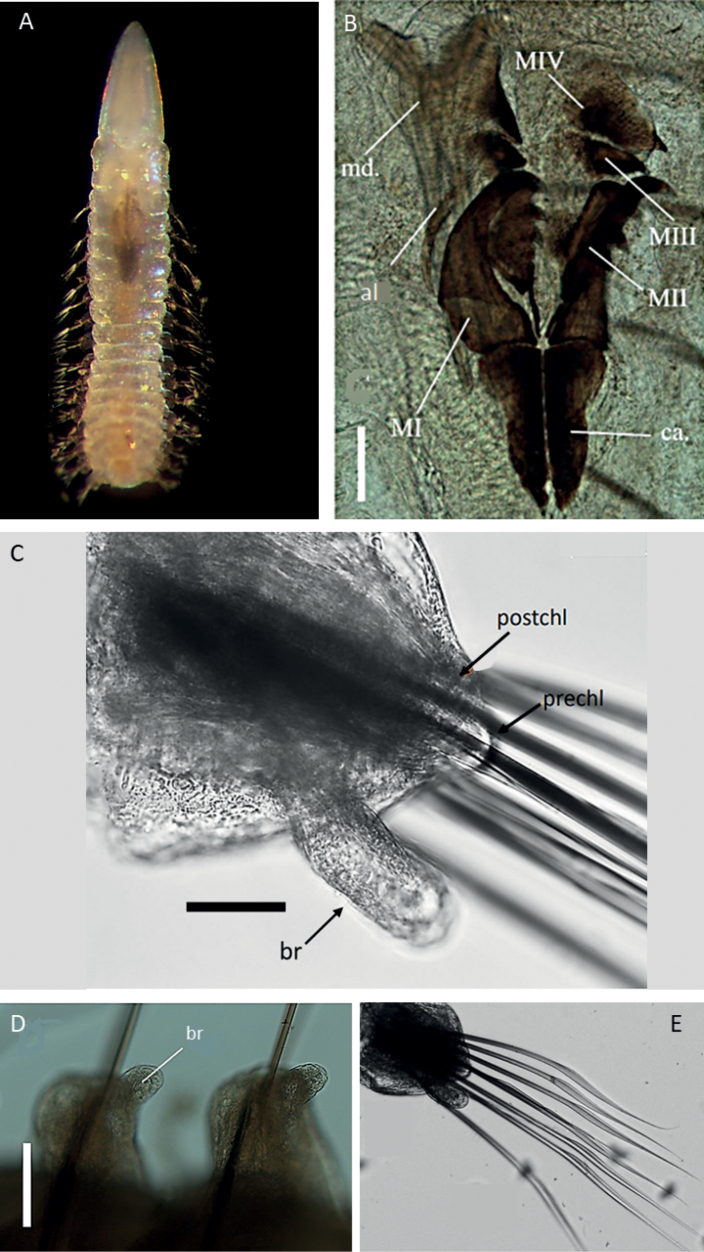
Lumbrineridae sp. NHM_1485 **A** live image of anterior fragment of specimen NHM_1516 in dorsal view **B** complete maxillary apparatus, specimen NHM_1516 **C** parapodium from chaetiger 10 in posterior view with prechaetal (prechl) and postchaetal (postchl) lobes and branchia (br) marked by arrows, specimen NHM_1485 **D** parapodia on chaetigers 19–20, with branchiae (br), specimen NHM_1485 **E** limbate capillaries from anterior chaetiger, specimen NHM_1485. Scale bars: 100 µm (**B**); 50 µm (**C, D**). Abbreviations: br = branchiae ca. = carriers, MI = maxilla 1, MII = maxilla 2, MIII = maxilla 3, MIV = maxilla 4, al = attachment lamellae.

Species represented by several posteriorly incomplete specimens. Voucher specimen NHM_1485, 7.5 mm long and 1 mm wide for 29 chaetigers long anterior fragment. Voucher specimen NHM_1516 (Fig. 4A) dissolved for jaws examination and now represented by jaws only (Fig. 4B). Molecular voucher NHM_1843 is a body fragment, identified by DNA only. Live specimen light pink colour (Fig. 4A), with iridescent sheen and faint spotted pattern across body and prostomium; a red/orange band can be seen along dorsum. Preserved specimens pale yellow in ethanol. The anterior end of the body thick, with a distinctive large collar.

Prostomium elongated, conical, and distally pointed, longer than wide (Fig. 4A).

Maxillary apparatus with four pairs of maxillae (Fig. 4B). All maxillae with attachment lamellae. MI forceps-like without internal accessory teeth. MI the same length as carriers and joined completely to the base. MII with ~ 3–5 distinctive teeth, and thin sclerotised ligaments on the posterior end, ~ 2/3 the length of MI. MIII completely pigmented. MIV approximately rectangular and unidentate. Mandibles fused for only ¼ of their length.

**Figure 5.**
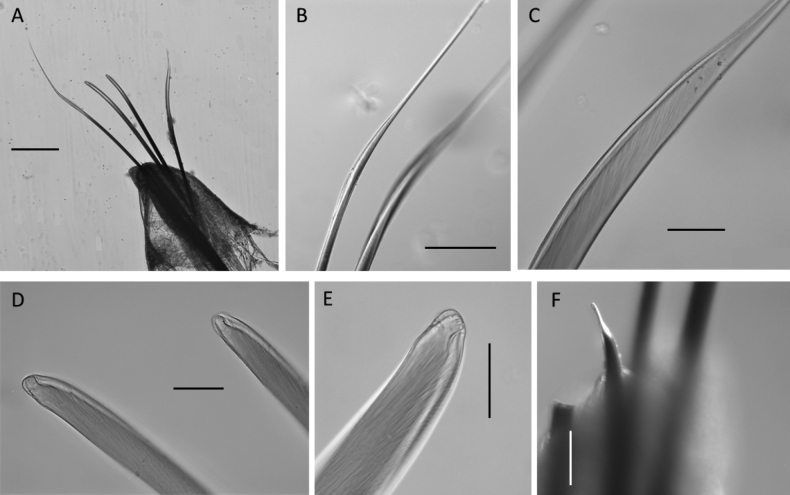
Lumbrineridae sp. NHM_1485, specimen NHM_1843 **A** parapodium from body fragment **B** limbate chaeta **C** detail of limbation **D** simple hooded hooks **E** detail of hooded hook dentition **F** acicula. Scale bars: 250 µm (**A**); 100 µm (**B**); 25 µm (**C–E**); 50 µm (**F**).

Parapodia consistent across body length, short and rounded. Pre-chaetal lobes broad and low in all chaetigers (Figs 4C, 5A). Branchiae present, simple, unbranched, elongated and digitiform (Fig. 4C), best developed in anterior chaetigers (Fig. 4C, D), at some point becoming absent (Fig. 5A).

Chaetae characterised by narrowly limbate capillaries (Figs 4E, 5A–C) and simple hooded hooks (Fig. 5A, D, E). Anterior fragment, specimen NHM_1485 with narrowly limbate capillaries only in 29 chaetigers long fragment. Chaetae increase in number from chaetigers 1–8, then decrease again from chaetiger nine onwards. From chaetiger 21, there are ~ 3 chaetae. Body fragment NHM_1483 with 1–3 narrowly limbate capillaries and one or two simple hooded hooks. Hooks long and slender, multidentate, with fine dentition of ~ 5 small teeth (Fig. 5D, E). Aciculae black (Fig. 4C, E), tips protruding (Fig. 5F). Posterior end and pygidium unknown.

##### Genetic data.

In our analysis, this species falls as a sister taxon to clade containing *Augeneria* species and unidentifiable CCZ specimen assigned to Lumbrineridae sp. NHM_2146 (Fig. 3). It has one COI match on GenBank with another unclassified annelid specimen, GenBank accession number KJ736520.1, also collected at the CCZ (Janssen et al. 2015).

##### Remarks.

We were not able to confidently identify CCZ specimens to the genus-level, as they were represented by two short anterior fragments (one now dissolved for jaws) and body fragment, identified by DNA only. No hooded hooks were observed in 29-chaetiger long anterior fragment of CCZ specimen NHM_1485, but they were present in all chaetigers on the body fragment of specimen NHM_1843, which was identified by DNA. Morphologically, elements of the maxillary apparatus (four pairs of dark maxillae, all with attachment lamellae, MII with ligaments) and chaetae composition (limbate chaetae and simple multidentate hooded hooks) are characteristic of several lumbrinerid genera. The digitiform structure associated with parapodia has been interpreted as branchia, pointing to the genus *Cenogenus* Chamberlin, 1919 (Oug pers. comms.). Due to the uncertainty of some characters such as hooks observed from the body fragment only (Fig. 5D–E), we identify this CCZ species to family level only and ascribe it to morphospecies Lumbrineridae sp. NHM_1485.

##### Distribution.

Central Pacific Ocean, Eastern CCZ, found in ‘UK-1’ and ‘OMS’ exploratory areas (Fig. 1).

#### ﻿ Augeneria

Taxon classificationAnimaliaEunicidaLumbrineridae

Monro, 1930

331A4570-BCE1-5088-BF98-591B73CE2D58

##### Type species.

*Augeneriatentaculata* Monro, 1930.

##### Diagnosis

(adapted from Orensanz 1973; Carrera-Parra 2006a; Oug et al. 2022). Prostomium with or without small antennae or nuchal papillae. Four pairs of maxillae, all with attachment lamellae. Maxillae I forceps-like. Maxillae II ca. as long as maxillae I, with ligament, without connecting plates. Maxillae III pigmented. Maxillae IV shaped like broad plates with whitish central and dark peripheral areas. Maxillae V absent. Mandible divergent at its anterior and posterior ends. Chaetae include limbate capillaries, simple and compound multidentate hooded hooks.

##### Remarks.

The predominantly deep-sea genus *Augeneria* Monro, 1930 has a confused taxonomic history. It was previously defined primarily by the presence of three occipital antenna as seen in the type species *A.tentaculata*, Monro 1930. Fauchald (1970) did not agree that this was enough to distinguish the genus from *Lumbrineris*, describing the antennae as eversible nuchal organs of “little generic significance”. Then, in his review, Orensanz (1973) revised *Augeneria* as a valid genus characterised by anterior pseudo-compound hooks, MII with three rounded teeth, and MIV with an expanded pale central area further de-emphasising the antennae in the diagnosis of the genus. *Augeneria* has since been redefined by Carrera-Parra (2006a) who presented a diagnosis of the genus that includes three occipital antennae and emphasis on attachment lamellae of the maxillary apparatus, a character that is barely mentioned in work by other authors. In the latest diagnosis given for this genus, Oug et al. (2022) recognised the antennae or nuchal papillae as either present or absent, to prevent several species currently referred to *Augeneria* being without generic affiliation. Therefore, it appears that currently there is no settled definition of *Augeneria*. In this paper, we present two species of *Augeneria*, and we primarily follow the definition with emphasis on form of MIV and chaetal composition as no antennae could be observed in any of our specimens.

Currently, the genus *Augeneria* includes eight valid species, mostly from deeper waters: *A.albidentata* (Ehlers, 1908) (originally described from Agulhas Bank, South Africa at 117 m), *A.algida* (Wirén, 1901) (from West Spitsbergen, Norway, Arctic Ocean at 1780 m), *A.bidens* (Ehlers, 1887) (from Florida and Cuba, Atlantic Ocean at 214–642 m), *A.polytentaculata* Imajima & Huguchi, 1975 (from Japan, Pacific Ocean at 100 m), *A.riojai* Aguirrezabalaga & Carrera-Parra, 2006 (from the Bay of Biscay, Atlantic Ocean at 480–580 m), *A.tentaculata* Monro, 1930 (from Signy Island, Antarctic Ocean at 244–344 m), *A.verdis* Hutchings & Murray, 1984 (from the Tasman Sea, Pacific Ocean at 4–12 m) and *A.profundicola* Kurt-Sahin, Çinar & Gonulal, 2016 (from Aegean Sea at 950 m). The validity of *A.dayi* within the genus *Augeneria* has been questioned, as it lacks compound hooded hooks on the parapodia and the original description by De Silva (1965) lacks a proper description of the morphology of the maxillary apparatus according to Kurt-Sahin et al. (2016). Furthermore, *Augeneriabidens* is currently listed on the WoRMS database as *Lumbrinerisbidens* (Read and Fauchald 2022a). No species of *Augeneria* has been described from abyssal depths to date.

#### ﻿ Augeneria

Taxon classificationAnimaliaEunicidaLumbrineridae

sp. NHM_4590

54A66982-831D-575C-B121-69EDBC97A72B

Figs 6A–G , 7A–F

##### Material examined.

NHM_0209, coll. 14 Oct. 2013, AB01, UK-1, Box core, 13.82412, -116.53425, 4054 m, https://data.nhm.ac.uk/object/1b024172-3aba-4404-9cd5-6f9cc86d69c0; NHM_0609, coll. 17 Feb. 2015, AB02, UK-1, EBS, 12.38624, -116.54867, 4202 m, https://data.nhm.ac.uk/object/98e583f4-665d-4197-8349-c8f6147454b8; NHM_0205, NHM ANEA 2022.832, coll. 14 Oct. 2013, AB01, UK-1, Box core, 13.82412, -116.53425, 4054 m, https://data.nhm.ac.uk/object/2af3e568-87be-4158-9b8e-f5b1c3cc5468; NHM_0686, NHM ANEA 2022.841, coll. 20 Feb. 2015, AB02, UK-1, EBS, 12.51317, -116.60417, 4425 m, https://data.nhm.ac.uk/object/1b1830d0-ec8f-4503-86ca-72efba0a4772; NHM_0782, NHM ANEA 2022.842, coll. 20 Feb. 2015, AB02, UK-1, EBS, 12.51317, -116.60417, 4425 m, https://data.nhm.ac.uk/object/10adaf27-6b76-4258-8530-5cb8ef631c44; NHM_0788, NHM ANEA 2022.843, coll. 20 Feb. 2015, AB02, UK-1, EBS, 12.51317, -116.60417, 4425 m, https://data.nhm.ac.uk/object/87c4f766-d47b-4bf4-85e3-b5e234cdf241; NHM_1008, NHM ANEA 2022.833, coll. 24 Feb. 2015, AB02, OMS, EBS, 12.13367, -117.292, 4122 m, https://data.nhm.ac.uk/object/9338cf46-ea85-43d8-9593-1d2395acb4ca; NHM_1872, NHM ANEA 2022.844, coll. 13 Mar. 2015, AB02, OMS, EBS, 12.0415, -117.21717, 4094 m, https://data.nhm.ac.uk/object/621a4712-aea8-42c6-8ad9-ee673f0d06c6; NHM_1878, NHM ANEA 2022.845, coll. 13 Mar. 2015, AB02, OMS, EBS, 12.0415, -117.21717, 4094 m, https://data.nhm.ac.uk/object/cd9d9fd6-547d-4b98-b111-d47e647333bf; NHM_1948K, NHM ANEA 2022.846, coll. 13 Mar. 2015, AB02, OMS, EBS, 12.0415, -117.21717, 4094 m, https://data.nhm.ac.uk/object/8e37077f-b5ff-4190-a75e-23dd07314798; NHM_2249, NHM ANEA 2022.836, coll. 1 Mar. 2015, AB02, OMS, EBS, 12.25733, -117.30217, 4302 m, https://data.nhm.ac.uk/object/d2bc88ee-e882-4f6d-aa4c-93bcdb429611; NHM_2389, NHM ANEA 2022.834, coll. 20 Feb. 2015, AB02, UK-1, EBS, 12.51317, -116.60417, 4425 m, https://data.nhm.ac.uk/object/5cd29524-9ddf-43a0-8618-5ec8f6dc0bf8; NHM_2588, NHM ANEA 2022.838, coll. 1 Mar. 2015, AB02, OMS, EBS, 12.25733, -117.30217, 4302 m, https://data.nhm.ac.uk/object/22fbc1a3-b2bd-40c2-bf7f-d3dcd6b85ea4; NHM_2976, NHM ANEA 2022.839, coll. 20 Feb. 2015, AB02, UK-1, EBS, 12.51317, -116.60417, 4425 m, https://data.nhm.ac.uk/object/efc8baac-defe-468f-a40d-5b0c2e160621; NHM_3886, NHM ANEA 2022.835, coll. 6 Mar. 2020, RC01, UK-1, Box core, 13.59013, -116.46817, 4081 m, https://data.nhm.ac.uk/object/10ae7a30-345f-40b0-9fb6-ec84eb4d91d5; NHM_4590, NHM ANEA 2022.837, coll. 15 Mar. 2020, RC01, OMS, Box core, 12.32636, -120.02542, 4157 m, https://data.nhm.ac.uk/object/4ed5a4c7-a44a-4cce-8e0b-4a5036fd5b5c; NHM_4738_ECDS4, NHM ANEA 2022.840, coll. 28 Feb. 2020, RC01, UK-1, Box core, 13.98698, -116.47664, 4059 m, https://data.nhm.ac.uk/object/fc4ac1a4-1a78-475f-a9bc-36740d2e1bd9.

##### Description.

**Figure 6.**
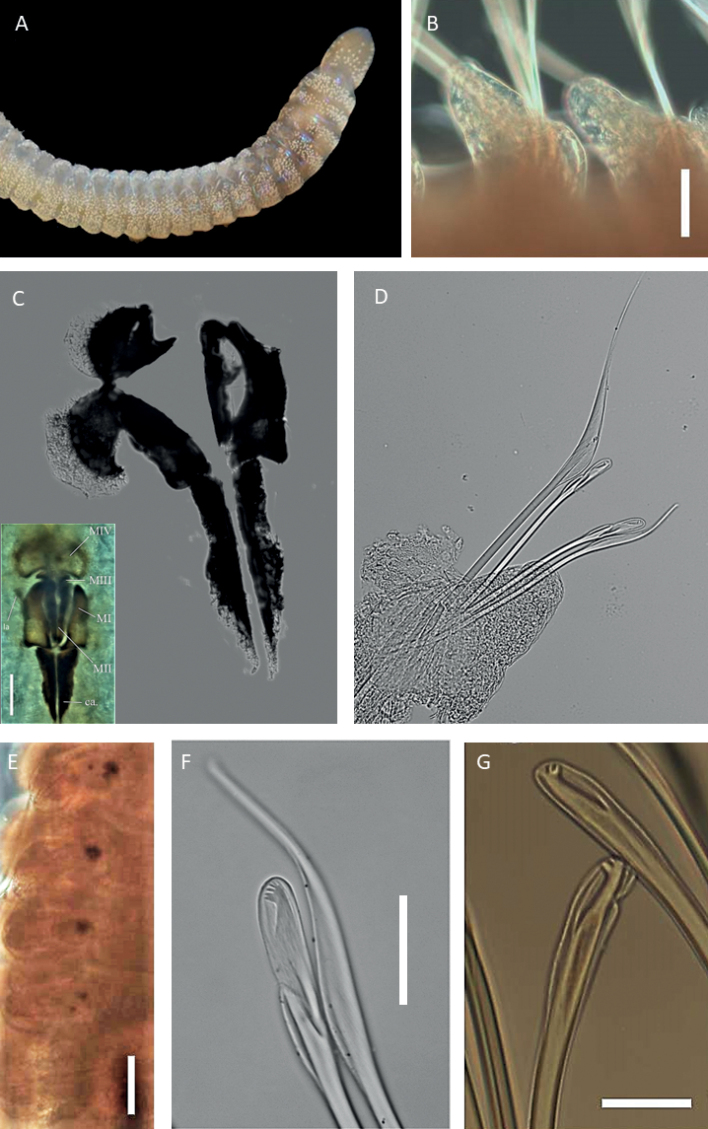
*Augeneria* sp. NHM_4590 **A** Live image of specimen NHM_0205 in lateral view **B** parapodia 5–7 (right to left), specimen NHM_1008 **C** complete maxillary apparatus specimen NHM_0205, inset - annotated image of the same taken in situ **D** compound multidentate hooded hooks and limbate capillary chaetae on chaetiger 4, specimen NHM_0205 **E** spotted parapodia pattern anterior specimen NHM_1008 **F** detail of compound multidentate hooded hook on chaetiger 4, specimen NHM_0205 **G** simple multidentate hooded hooks on chaetiger 30 specimen NHM_3886. Scale bars: 50 µm (**B**); 100 µm (**C, E**); 25 µm (**F, G**). Abbreviations: ca. = carriers, MI = maxilla 1, MII = maxilla 2, MIII = maxilla 3, MIV = maxilla, al = attachment lamellae.

**Figure 7.**
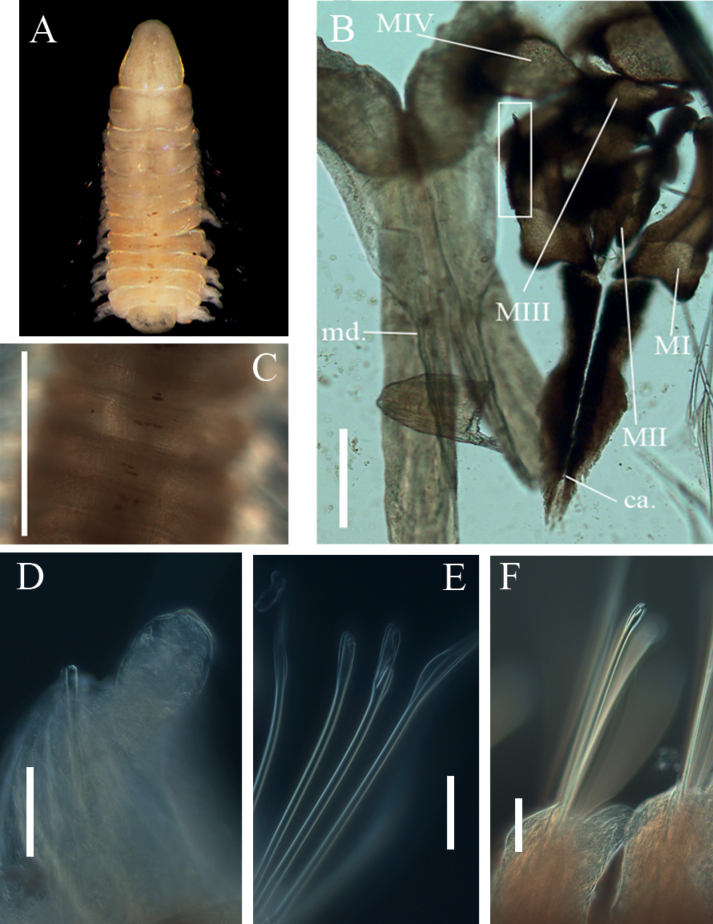
*Augeneria* sp. NHM_4590 **A** anterior end of live specimen NHM_2249 in dorsal view **B** complete maxillary apparatus specimen NHM_2249 (attachment lamella of MI indicated by rectangle) **C** spotted pattern on dorsum, specimen NHM_2249 **D** posterior globular post-chaetal lobe, specimen NHM_2249 **E** simple multidentate hooded hook and compound multidentate hooded hook on chaetiger 8, specimen NHM_2249 **F** posterior simple multidentate hooded hook specimen NHM_4590. Scale bars: 100 µm (**B**); 500 µm (**C**); 50 µm (**D, E, F**). Abbreviations: ca. = carriers, MI = maxilla 1, MII = maxilla 2, MIII = maxilla 3, MIV = maxilla 4, md. = mandibles.

Species represented by complete specimen NHM_4590 and several posteriorly incomplete specimens. Voucher specimen NHM_4590 in two fragments, anterior fragment 5.5 mm and 0.85 mm wide for 33 chaetigers, posterior fragment 8 mm long for ~ 50 chaetigers. Voucher specimen NHM_0205 (Fig. 6A), 2.3 mm long and 0.5 mm wide for 14 chaetigers long anterior fragment. Voucher specimen NHM_2249 (Fig. 7A) represented by body fragment and jaws only as anterior end tissues dissolved for jaws observation (Fig. 7B). Live specimen pale yellow to translucent, with distinct white spotted pattern across each chaetiger (Fig. 6A), spotted pattern also on prostomium in two triangular peaks along the ventral side and lateral edge. Pattern lost in specimens preserved in ethanol; some larger specimens with yellow-orange tint when preserved in ethanol, smaller specimens appear white; slight red pigmentation runs down the dorsal side of the body in some specimens e.g., NHM_2249 (Fig. 7A, C). Body wide anteriorly tapering slightly towards posterior, chaetigers becoming more bead-like towards posterior.

Prostomium broadly conical, distally rounded, ca. as long as wide (Fig. 6A), with a spotted pattern that is slightly visible when preserved in ethanol, prostomium can also appear slightly pear-shaped (Fig. 7A).

Maxillary apparatus with four pairs of maxillae, central areas non-pigmented, with dark edges (Figs 6C, 7B). All maxillae with attachment lamellae. MI and MIV appear darker around the edges. MI with enlarged base that connects with carriers, though overlapping the edge of them. MI forceps-like, slender and hooked towards posterior end. Carriers pointed with a lateral incision and are equal in length to MI. MII with ~ 3 teeth, with short ligaments. MIII small, darker along anterior lateral edge. MIV large and oval shaped spanning the width of the maxillary apparatus, with a dark edge and pale interior. Mandibles fused along ¾ of length, slightly divergent at both ends (Fig. 7B).

Parapodia uniramous, large, and distinct (Fig. 6B, D). Pre-chaetal lobe small and rounded. Postchaetal lobe elongated, digitiform, pointing towards the posterior of the body almost parallel from parapodia 1–9 after which the base of the parapodia becomes wider and the lobes begin to point away from the body. Posterior postchaetal lobes appear globular and reduced (Fig. 7D). Darker spots of colouration at the base of parapodia.

Chaetae characterised by limbate capillaries, compound multidentate hooded hooks and simple multidentate hooded hooks. Chaetigers 1–8 with ca. two compound multidentate hooded hooks and limbate chaetae (Figs 6D, 7E–F). In some specimens, chaetiger 8 with one simple and one compound multidentate hooded hook. Compound multidentate hooks with short blades, with ~ 6 small teeth in lateral view (Fig. 6F). Chaetiger 9 onwards with 2–4 simple multidentate hooded hooks only (Fig. 6G). Aciculae yellow. Posterior chaetigers with two simple multidentate hooded hooks only (Fig. 7F).

Pygidium observed in posterior fragment of specimen NHM_4590, with four short, subdistally inserted, distally narrowing cirri.

##### Genetic data.

This species falls within a well-supported monophyletic clade containing *Augeneria* species, another CCZ species included in this paper - *Augeneria* sp. NHM_0851 and unidentifiable CCZ specimen Lumbrineridae sp. NHM_2146 (Fig. 3). There is one COI match to this species on GenBank with an unassigned species also collected from the CCZ, GenBank accession number KJ736519.1 (Janssen et al. 2015).

##### Remarks.

This species can be varied in appearance, for example specimen NHM_2249 (Fig. 7A) has a pear-shaped prostomium, whereas in specimen NHM_0205 it is rounded (Fig. 6A). Additionally, variations in patterning and colouration have been observed (Figs 6E, 7C), with several specimens having a prominent orange colouration in the anterior. Nevertheless, genetic data identified only one species, so the observed variability is best explained as intraspecific. The form of the hooks has been interpreted as compound, but they may approach the pseudo-compound form with the slit apparently being closed at one side (Oug, pers. comms.).

The maxillary apparatus and chaetae composition of this species are indicative of the genus *Augeneria* Monro, 1930. Molecular data also support assignment of this species to genus *Augeneria* (Fig. 3). This CCZ species resembles *Augeneriabidens* (Ehlers, 1887) based on re-description by Carrera-Parra (2001), who examined the type specimens. The type locality for *Augeneriabidens* is in the Gulf of Mexico and Caribbean Sea in depths of 214–348 m (original description as *Lumbriconerisbidens* Ehlers, 1887). It has also been documented in Maryland to North Carolina in the US waters (Fauchald et al. 2009). The maxillary apparatus is described by Carrera-Parra (2001) as follows; carriers shorter than MI and rounded anteriorly; well-developed attachment lamellae; MII with three rounded teeth; MIII and MIV with pale central and dark peripheral areas (Carrera-Parra 2001). Compound hooks have a similar distribution as in CCZ specimen by being present between chaetigers 1 and 7–15, with simple hooks present from chaetigers 8–16 (Carrera-Parra 2001). Carrera-Parra remarks that the position of transition between compound and simple hooks is size dependant. However, the CCZ species can be distinguished by the form of MIV, which is semi-circular (Figs 6C, 7B) rather than square-shaped as in *A.bidens* and by having much longer mandibles (Fig. 7B). No antennae were observed in CCZ specimens. Lastly, *A.bidens* has been described from much shallower depths (214–348 m) compared to ~ 4500 m for the CCZ species. Given that no *Augeneria* species have been described from the abyssal depths to date, the CCZ specimens likely represent a new species, but further taxonomic work will be necessary. Currently, we assign the CCZ specimens to morphospecies *Augeneria* sp. NHM_4590.

##### Distribution.

Central Pacific Ocean, Eastern CCZ, found in ‘UK-1’, ‘OMS’ and ‘NORI-D’ exploratory areas (Fig. 1).

#### ﻿ Augeneria

Taxon classificationAnimaliaEunicidaLumbrineridae

sp. NHM_0851

758EAAC7-B277-59A9-BDD6-A80BCB9AC542

Fig. 8A–G

##### Material examined.

NHM_0420, coll. 20 Oct. 2013, AB01, UK-1, ROV, 13.86367, -116.54432, 4011 m, https://data.nhm.ac.uk/object/bde33da0-8f07-470a-b63a-979326313974; NHM_0737, NHM ANEA 2022.850, coll. 20 Feb. 2015, AB02, UK-1, EBS, 12.51317, -116.60417, 4425 m, https://data.nhm.ac.uk/object/a9119f6d-0787-40a4-87cd-08cc80d8b35b; NHM_0761, NHM ANEA 2022.847, coll. 20 Feb. 2015, AB02, UK-1, EBS, 12.51317, -116.60417, 4425 m, https://data.nhm.ac.uk/object/52a58501-7bfc-4dcc-af95-0509a6463600; NHM_0851, NHM ANEA 2022.848, coll. 21 Feb. 2015, AB02, UK-1, Box core, 12.57903, -116.72378, 4218 m, https://data.nhm.ac.uk/object/71820a17-178d-4fcd-b493-8fac5f8b45cb; NHM_2441, NHM ANEA 2022.849, coll. 17 Feb. 2015, AB02, UK-1, EBS, 12.38624, -116.54867, 4202 m, https://data.nhm.ac.uk/object/a0b38651-82c1-4410-a352-33da931260ad.

##### Description.

**Figure 8.**
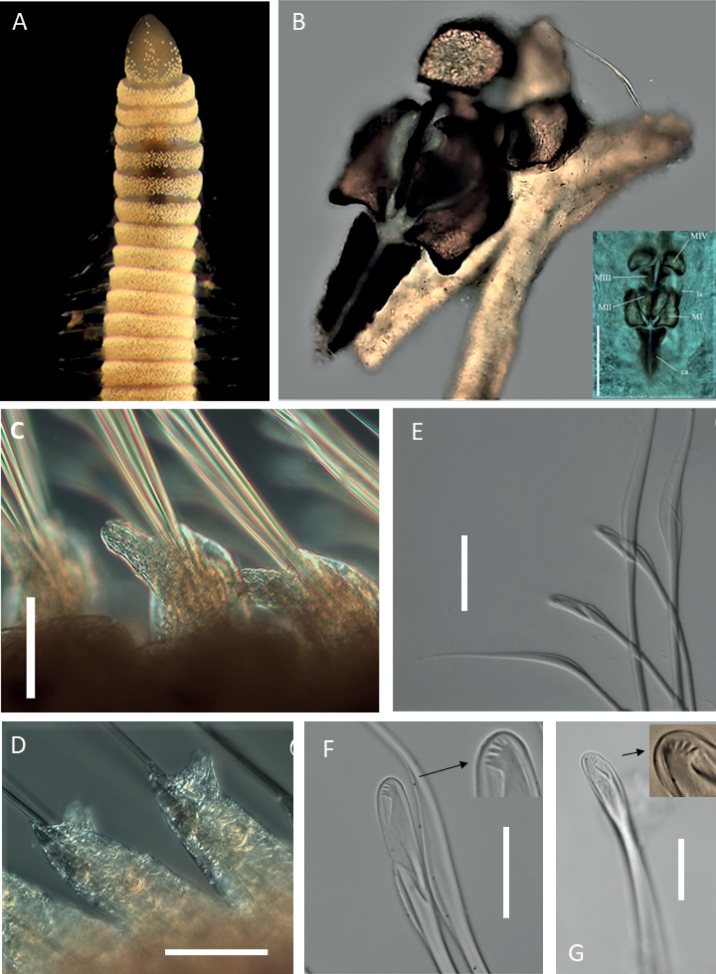
*Augeneria* sp. NHM_0851 **A** live specimen NHM_0851, anterior end in dorsal view **B** complete maxillary apparatus, insert – annotated in situ image of the same, specimen NHM_0420 **C** parapodium 7 with well-developed postchaetal lobe, specimen NHM_0851 **D** parapodia 29 and 30, specimen NHM_0851 **E** compound multidentate hooded hooks and limbate chaetae from anterior chaetiger **F** compound multidentate hooded hook on chaetiger 9, specimen NHM_0851 **G** simple multidentate hooded hook chaetiger 10, specimen NHM_0851. Scale bars: 125 µm (**B** insert); 100 µm (**C, D**); 50 µm (**E**); 25 µm (**E, F, G**). Abbreviations: ca. = carriers, MI = maxilla 1, MII = maxilla 2, MIII = maxilla 3, MIV = maxilla 4, al. = attachment lamellae.

Species represented by several posteriorly incomplete specimens. Voucher specimen NHM_0851 (Fig. 8A), 5.8 mm long and 0.75 mm wide for 34 chaetigers long anterior fragment.

Live specimen with translucent iridescent colouration and distinctive white spotted pattern across chaetigers and prostomium (Fig. 8A), with red spots at each parapodium and faint central line running down the ventral side of the body. Preserved specimens milky white to yellow with parapodial spots visible.

Prostomium conical, wide at base, distally narrowing, ca. as long as wide (Fig. 8A). Peristomium prominent forming wide collar (Fig. 8A).

Maxillary apparatus with four pairs of maxillae, central areas non-pigmented, with dark edges (Fig. 8B). All maxillae with attachment lamellae. MI ca. equal length to carriers, with wide base on the anterior end that overlaps. MII slightly shorter or equal length than MI, with ligaments. MIV forms a squarish plate with dark edges and a pale centre. Mandibles fused for ~ ½ their length.

Parapodia in anterior wider and shorter with small rounded pre-chaetal lobes and long digitiform postchaetal lobes (Fig. 8C), in the posterior the base becomes elongated and postchaetal lobes become significantly shorter and conical (Fig. 8D).

Chaetae characterised by limbate capillaries, compound multidentate hooded hooks and simple multidentate hooded hooks. Capillaries long and slender, narrowly limbate (Fig. 8E). Chaetigers 1–11 with two or three compound multidentate hooded hooks and winged capillaries, except for chaetiger 5 which has four hooks. Compound multidentate hooks with short blades, with ~ 6 small teeth in lateral view (Fig. 8F). Chaetiger 10 with one simple multidentate hooded hook, and two compound multidentate hooded hooks. Chaetiger 11 onwards with simple multidentate hooded hooks along with limbate chaetae. Simple hooks with ~ 6 small teeth in lateral view (Fig. 8G). Chaetiger 25 onwards, with two simple multidentate hooded hooks only. Aciculae yellow. Posterior and pygidium unknown.

##### Genetic data.

This species falls within a well-supported monophyletic clade containing *Augeneria* species, another CCZ species included in this paper - *Augeneria* sp. NHM_4590 and unidentifiable CCZ specimen Lumbrineridae sp. NHM_2146 (Fig. 3).

##### Remarks.

Chaetae composition and maxillary apparatus are indicative of the genus *Augeneria* Monro, 1930. Similarly, to *Augeneria* sp. NHM_4590, form of the hooks has been interpreted as compound, but they may approach pseudo-compound form with the slit apparently being closed at one side (Oug, pers. comms.) This species was numerous within our samples. However, we were unable to match the description with any known species. It can be distinguished from *Augeneria* sp. NHM_4590 also found in CCZ samples by mainly the shape of MIV and MII. In *Augeneria* sp. NHM_0851 MIV are more squarish (Fig. 8B) rather than semi-circular (Figs 6C, 7B) and MII has rounded rather than pointed teeth as in *Augeneria* sp. NHM_4590. This species also shares some characters with *Augeneriaverdis* Hutchings & Murray, 1984, though it differs in the lack of obvious green colouration. Additionally, *Augeneria* sp. NHM_0851 has carriers with a distinct lateral incision, whereas the carriers in *Augeneriaverdis* are described as triangular with a “shallow” incision.

Given that no *Augeneria* species have been described from the abyssal depths to date, the CCZ specimens likely represent a new species, but further taxonomic work will be necessary. Currently, we assign the CCZ specimens to morphospecies *Augeneria* sp. NHM_0851.

##### Distribution.

Central Pacific Ocean, Eastern CCZ, found in ‘UK-1’, ‘OMS’ and ‘NORI-D’ exploratory areas (Fig. 1).

#### ﻿ Lumbrinerides

Taxon classificationAnimaliaEunicidaLumbrineridae

Orensanz, 1973

02FBA27A-9BCE-569F-A717-96A0ACE203CD

##### Type species.

*Lumbrineridesgesae* Orensanz, 1973.

##### Diagnosis

(based on Miura 2017). Body cylindrical without colour pattern; prostomium acorn-shaped with tapered distal end, pygidium with semi-circular profile. Maxillary apparatus comprising four pairs of maxillae; maxilla I furcate with or without accessory teeth on inner edge, maxilla II a semi-circular plate with two or three teeth, maxilla III a rectangular (semi-circular in dorsal view) plate lacking well-formed teeth on cutting edge, maxilla IV a long broad oval plate without obvious teeth; maxillary carriers long, thick, winged posteriorly; lateral supports triangular comprising many thin small plates. All species with limbate chaetae and simple bidentate hooded hooks.

#### ﻿ Lumbrinerides cf. laubieri

Taxon classificationAnimaliaEunicidaLumbrineridae

(NHM_0020)

38FF7311-7E66-5EDB-B5E0-412309984A19

**Figure 9.**
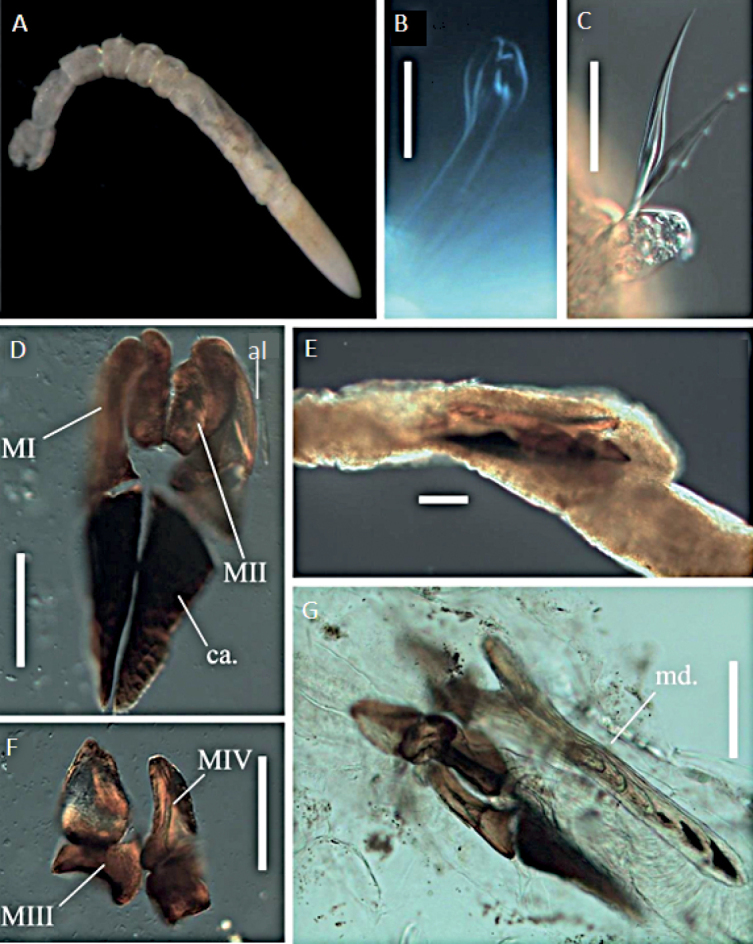
Lumbrineridescf.laubieri**A** anterior fragment of specimen NHM_1146 in dorsal view **B** simple bidentate hooded hook on chaetiger 3, specimen NHM_1146 **C** winged limbate capillary chaetae on chaetiger 5 specimen, NHM_1146 **D** maxillary apparatus, specimen NHM_1146 E everted jaws in specimen NHM_3492 **F** maxillary apparatus of specimen NHM_1146 (MIII = maxilla 3, MIV = maxilla 4) **G** maxillary apparatus of specimen NHM_2245 (md. = mandibles). Scale bars: 50 µm (**C**); 10 µm (**B**); 100 µm (**D, E, F, G**). Abbreviations: ca. = carriers, MI = maxilla 1, MII = maxilla 2, al = attachment lamellae.

**Figure 10.**
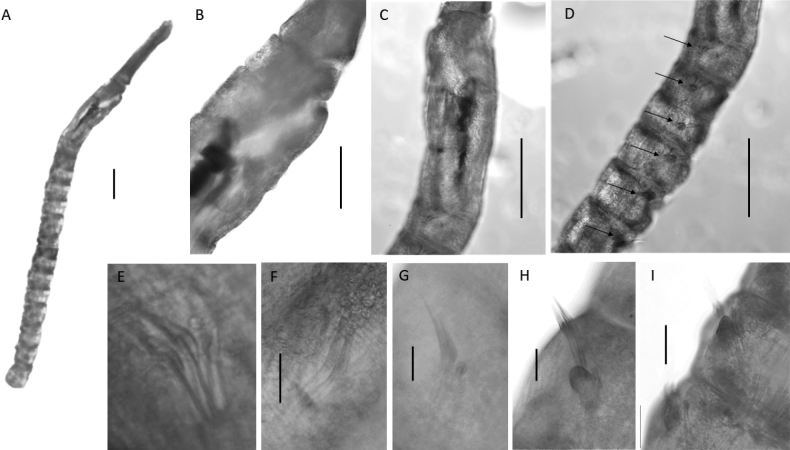
Lumbrineridescf.laubieri (specimen NHM_ 4378_ECDS5) **A** posteriorly incomplete preserved specimen **B** anterior end with margins between prostomium and 2-rigned peristomium **C** anterior end with margins between prostomium, peristomium and chaetiger 1 **D** chaetigers 2–7 marked by arrows, showing the shift of parapodia from lateral to dorsal position **E** chaetiger 1 **F** chaetiger 2 **G** chaetiger 3 **H** chaetiger 4 with postchaetal lobe **I** chaetigers 5–6 with postchaetal lobes. Scale bars: 250 µm (**A, C, D**); 100 µm (**B**); 25 µm (**F, G, H**); 50 µm (**I**).

**Figure 11.**
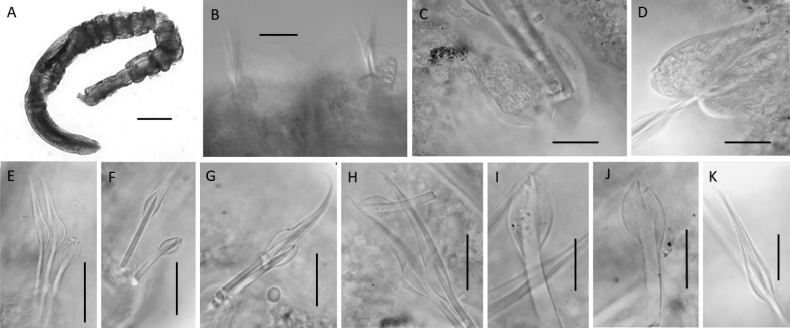
Lumbrineridescf.laubieri (specimen NHM_0020) **A** posteriorly incomplete preserved specimen **B** parapodia of chaetiger 4 and dorsally shifted parapodia of chaetiger 5, both with developed postchaetal lobes **C** detail of pre- and postchaetal lobes of chaetiger 4 **D** detail of pre- and postchaetal lobes of chaetiger 5 **E** chaetae of chaetiger 1, with limbate chaetae showing broad limbation (elbow) **F** chaetae of chaetiger 2 **G** chaetae of chaetiger 3 **H** chaetae of chaetiger 4 **I** simple hooded hook of chaetiger 4 **J** simple hooded hook of chaetiger 7 **K** narrowly limbate capillary from chaetiger 5. Scale bars: 250 µm (**A**); 50 µm (**B**); 25 µm (**C–K**).

Figs 9A–G , 10A–I , 11A–K

##### Material examined.

NHM_0020, NHM ANEA 2022.801, coll. 9 Oct. 2013, AB01, UK-1, EBS, 13.8372, -116.55843, 4336 m, https://data.nhm.ac.uk/object/cfc84885-578e-4b87-a14f-e8fc1cf2a5a0; NHM_0028, NHM ANEA 2022.802, coll. 9 Oct. 2013, AB01, UK-1, EBS, 13.8372, -116.55843, 4336 m, https://data.nhm.ac.uk/object/0adcf12b-4027-4893-98a2-588d60e352a3; NHM_1146, NHM ANEA 2022.803, coll. 26 Feb. 2015, AB02, OMS, EBS, 12.1155, -117.1645, 4100 m, https://data.nhm.ac.uk/object/4849b790-53a1-45f8-a7e0-3f60062642cd; NHM_2245, NHM ANEA 2022.805, coll. 1 Mar. 2015, AB02, OMS, EBS, 12.25733, -117.30217, 4302 m, https://data.nhm.ac.uk/object/024a6d28-6775-4d01-823b-3b0a03fcd727; NHM_3492, NHM ANEA 2022.804, coll. 1 Mar. 2020, RC01, OMS, Box core, 14.03696, -116.50802, 4138 m, https://data.nhm.ac.uk/object/e6b00cf4-5c01-453d-a1ff-5c7dc0783960; NHM_4738_ECDS1, NHM ANEA 2022.808, coll. 28 Feb. 2020, RC01, UK-1, Box core, 13.98698, -116.47664, 4059 m, https://data.nhm.ac.uk/object/c78086ef-af8a-4b1c-b9db-e61df25585f4; NHM_4738_ECDS3, NHM ANEA 2022.807, coll. 28 Feb. 2020, RC01, UK-1, Box core, 13.98698, -116.47664, 4059 m, https://data.nhm.ac.uk/object/7d7e9048-38fa-4503-b256-6e6ac3c23687; NHM_4738_ECDS5, NHM ANEA 2022.806, coll. 28 Feb. 2020, RC01, UK-1, Box core, 13.98698, -116.47664, 4059 m, https://data.nhm.ac.uk/object/2806a25b-e00e-4845-9b6a-10ca95b3fd6a; NHM_4743_ECDS1, NHM ANEA 2022.809, coll. 4 Mar. 2020, RC01, UK-1, Box core, 13.99732, -116.52824, 4102 m, https://data.nhm.ac.uk/object/d42c9eb3-2052-4e90-9e00-61e20291d674; NHM_8777_HW01, NHM ANEA 2022.811, coll. 12 Nov. 2020, DG05a, NORI-D, Box core, 10.3781, -117.14689, 4300 m, https://data.nhm.ac.uk/object/18c5a2d0-2bd6-48e2-bd02-9fe7c03ae7a4; NHM_8810, NHM ANEA 2022.815, coll. 23 Nov. 2020, DG05a, NORI-D, Box core, 10.3554, -117.22087, 4289 m, https://data.nhm.ac.uk/object/75d2356a-cfd2-4ea8-b6d4-7d69ee27cb1c; NHM_8855, NHM ANEA 2022.816, coll. 30 Oct. 2020, DG05a, NORI-D, Box core, 10.92904, -116.26351, 4262 m, https://data.nhm.ac.uk/object/2250d689-4b91-4998-8776-05db9d437c89; NHM_8874, NHM ANEA 2022.817, coll. 2 Nov. 2020, DG05a, NORI-D, Box core, 10.9714, -116.16494, 4240 m, https://data.nhm.ac.uk/object/4d437d57-72b5-4f7a-84b7-87b13b6c422d; NHM_8898_LN01, NHM ANEA 2022.812, coll. 2 Nov. 2020, DG05a, NORI-D, Box core, 10.97448, -116.35427, 4260 m, https://data.nhm.ac.uk/object/af52e52a-9c50-432e-a88c-1f03cb6f981c; NHM_8898_LN02, NHM ANEA 2022.813, coll. 2 Nov. 2020, DG05a, NORI-D, Box core, 10.97448, -116.35427, 4260 m, https://data.nhm.ac.uk/object/123e1f28-a76e-4929-9c58-7ffd80db14a2; NHM_8898_LN03, NHM ANEA 2022.814, coll. 2 Nov. 2020, DG05a, NORI-D, Box core, 10.97448, -116.35427, 4260 m, https://data.nhm.ac.uk/object/2c462f72-683e-4fb3-bef9-8a8fe10b98f3; NHM_8798_HW02, NHM ANEA 2022.810, coll. 8 Nov. 2020, DG05a, NORI-D, Box core, 10.32571, -117.17753, 4300 m, https://data.nhm.ac.uk/object/568f9a43-2c36-49be-8829-55edb69ba271.

##### Comparative material examined.

**Figure 12.**
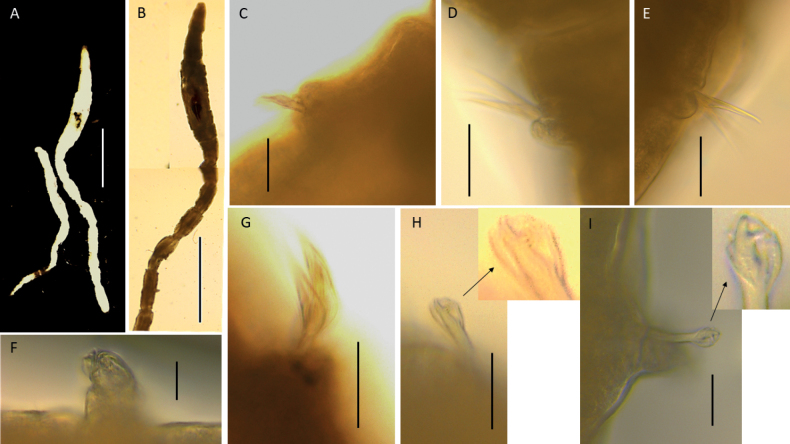
*Lumbrinerideslaubieri*, holotype MNHN.1278 **A, B** preserved specimen in dorsal view **C** chaetiger 1 **D, E** chaetiger 4 with postchaetal lobe **F** postchaetal lobe from chaetiger 10 **G** broadly limbate chaetae from chaetiger 1 **H, I** hooded hooks, with inserts showing the detail of bidentate dentition. Scale bars: 1 mm (**A, B**); 250 µm (**C, F G, H, I**); 500 µm (**D, E**).

Fig. 12A–I. *Lumbrinerideslaubieri* Miura, 1980; holotype MNHN.1278. NE Atlantic, Gulf of Gascogne, Biogas IV, DS61, coll. 24 Feb 1974, 24.02.1974, 47.5686111, -9.6355556, 2 250 m.

##### Description.

All specimens posteriorly incomplete, including voucher specimens NHM_0020, NHM_ 4378_ECDS5, NHM_1146, NHM_2245 and NHM_3492. Body slender, narrow, and cylindrical measuring up to 3.1 mm in length for 11 chaetigers and width of 0.2–0.25 mm. Live specimens iridescent and slightly translucent, with visible spotted pattern along sides of prostomium; preserved specimens milky white in ethanol (Fig. 9A). Jaws typically visible through body (Fig. 9E).

Prostomium significantly longer than wide (Figs 9A, 10A, 11A), narrow, conical, bluntly pointed, with small papilla at the tip; with spotted pigmentation across length of prostomium. Peristomium with two rings (Fig. 10B, C), the first with a V-shaped notch on the ventral side.

Maxillary apparatus with four pairs of maxillae (Fig. 9D, F). All maxillae with attachment lamellae. Carriers as long as MI and joined to their entire base (Fig. 9D). Carriers with wide anterior base and coloured dark brown to black. MI without internal accessory teeth. Attachment lamellae along lateral edge of MI, thin and weakly sclerotised. MII shorter than MI with three rounded teeth. MIII as edentate plate, pigmented dark brown. MIV as edentate plate, appear elongated and slightly pointed, approximately triangular (Fig. 9F). Mandibles fused for entire length with three pigmented bands (Fig. 9G).

Parapodia reduced to. Chaetigers 1 and 2 distinctly longer than wide, with reduced parapodia, no lobes developed, situated laterally (Fig. 10D, E, F). Chaetiger 3 ca. as wide as long, with reduced parapodia shifted dorsolaterally, no lobes developed (Fig. 10D, G). Chaetiger 4 ca. as wide as long with small tongue-like postchaetal lobe (Figs 10D, H, 11C), shifted dorsolaterally. From chaetiger 5, chaetigers ca. as wide as long, shifted dorsally, with small tongue-like postchaetal lobes developed (Figs 10D, I, 11B, D). Pre-chaetal lobes always smaller than postchaetal lobes.

Chaetae of two types observed in all chaetigers: 1. simple bidentate hooded hooks with two teeth at ~ 45° from each other, with subdistal spur, 1–3 hooks per parapodium, usually two present (Figs 9B, 11I, J) and two winged limbate capillaries (Fig. 9C), usually two per parapodium; in chaetiger 1–4 capillaries broadly limbate (with elbow) (Fig. 11E–H), from chaetiger 5 narrowly limbate (Figs 9C, 11K). Aciculae yellow. Remainder of body and pygidium unknown.

##### Genetic data.

In our phylogenetic analysis, Lumbrineridescf.laubieri (NHM_0020) forms a well-supported monophyletic clade with another, as yet unnamed *Lumbrinerides* species (Fig. 3).

There are many identical or near-identical COI matches to unnamed specimens on GenBank that were previously collected at the CCZ (Janssen et al. 2015, 2019). There are no genetic data from the type or non-type specimens of *Lumbrinerideslaubieri*.

##### Remarks.

This small species was the most abundant lumbrinerid in our CCZ samples, represented by 211 specimens. Morphologically, this species is similar to *Lumbrinerideslaubieri* Miura, 1980, described from the Gulf of Gascony, France at lower bathyal depths of 1894–2775 m. Outside its type locality, *L.laubieri* has been reported in the North Aegean Sea at 156–300 m (Simboura and Zenetos 2005). Following the initial examination of CCZ specimens, these matched *L.laubieri* in several instances: small body size; greatly elongated prostomium; reduced parapodia in first three chaetigers; maxillae I without accessory teeth; mandibles with “concentric striations” consistent with the coloured bands seen on CCZ specimens (Fig. 9G); attachment lamellae supporting the maxillary apparatus present (Fig. 9D); two types of chaetae present, limbate capillary chaetae and simple bidentate hooded hooks (Fig. 9B, C).

The holotype MNHN.1278 of *L.laubieri* was re-examined as part of this study (Fig. 12A–I). The holotype is a small, very slender specimen, consisting of three fragments: the anterior fragment with 15 chaetigers being 4.5 mm long and 0.25 mm wide (Fig. 12A, B), a body fragment with six chaetigers, and a small 2-chaetiger long body fragment. The jaws were partially damaged during a previous investigation. Chaetae (particularly the limbate type) were often broken off.

In a recent revision of *Lumbrinerides* from Japanese water, Miura (2017) suggested new characters of taxonomic importance in this genus. Therefore, during the examination of type material particular attention has been paid to the following characters: the chaetiger on which the first hooks arise, the number of hooks per parapodium and the number of anterior reduced parapodia. No obvious differences were observed (Table 2), other than the presence of up to three hooks in some parapodia in CCZ specimens, while at most two were observed in the holotype of *L.laubieri*. However, other more subtle differences were observed that are usually not considered in the discussion of the taxonomic characters in Lumbrineridae. These differences refer to relative size of various features, unlikely to be related to size of specimen as all individuals investigated had small and very slender body of similar dimensions. The hooded hooks in CCZ specimens are very small and slender, with dentition only clearly observable under oil (x100 magnification), while the hooks in *L.laubieri* are much chunkier and easy to observe even under lower magnification (× 40) (Fig. 12H, I). Similarly, the development of broad limbation (elbow) on capillaries of anterior chaetigers is much more distinct in *L.laubieri* (Fig. 12C, G) compared to CCZ specimens (Fig. 12E). Characters discussed by Miura (2017) or in this study are summarised in Table 2.

**Table 2.** Comparison of selected characters of *L.laubieri* and CCZ specimens.

Character	Holotype MNHN.1278	CCZ specimens
chaetiger on which the first hooks arise	1	1
the number of hooks per chaetiger	1–2	1–3 (mostly 2)
dentition of the bidentate hook	chunky (easily observed under ×40 mag); teeth separated by ~ 90°	slender (need to be observed under ×100 mag); teeth separated by ~ 45°
the number of limbate chaetae per chaetiger	2?	1–2
limbate chaetae of anteriormost chaetigers	broad elbow well developed	broad elbow less developed
the number of chaetigers with reduced parapodia (=no lobes developed)	1–3	1–3
parapodia inserted laterally	on ch. 1–2	on ch. 1–2
parapodia with dorsolateral position	?	ch 3–4
parapodia inserted dorsally	from chaetiger 5	from chaetiger 5

Lastly, a bathymetric distributional pattern should be also taken into consideration as *L.laubieri* has been found in shallower depth (1894–2775 m) in the Atlantic, compared to CCZ specimens (~ 5000 m) in the Pacific. Depth is considered to be a greater barrier to gene flow compared to with horizontal distances (e.g., Atlantic vs. Pacific) (Taylor and Roterman 2017). Unfortunately, molecular data from *L.laubieri* is not available for comparison. Thus, it is currently difficult to establish the new species based on CCZ specimens, and these are cautiously ascribed to Lumbrineridescf.laubieri Miura, 1980.

Two more *Lumbrinerides* species have a conical, greatly elongated prostomium: *Lumbrineridescarpinei* (Ramos, 1976) described from Mediterranean Sea (off Monaco, at depths of 200–600 m) and *Lumbrineridesyoshioi* Miura, 2017 from shallow depths off Hokkaido, Japan. However, they have the following differences from CCZ specimens. *Lumbrineridescarpinei* has one long apodous segment rather than two peristomial rings, lacks visible mandibles and has accessory tooth on MI (Miura 1980). *Lumbrineridesyoshioi* differs in having 9–10 reduced anterior parapodia (as opposed to 3 in CCZ specimens) and in MI having two weakly projected accessory teeth (as opposed to no accessory teeth in CCZ specimens).

##### Distribution.

Central Pacific Ocean, Eastern CCZ, found in ‘UK-1’, ‘OMS’ and ‘NORI-D’ exploratory areas (Fig. 1).

#### ﻿ Lumbrineris

Taxon classificationAnimaliaEunicidaLumbrineridae

de Blainville, 1828

AEE9D39C-D7D2-5FBA-9720-54C288AF8C09

##### Type species.

*Nereisebranchiata* Pallas, 1788

##### Diagnosis

(after Carrera-Parra (2006b)). Prostomium without antennae, without eyes. Maxillary apparatus with five pairs of maxillae, carriers as long as MI, joined along entire base of MI; MI forceps-like without inner accessory teeth, with attachment lamella; MII as long as MI, with ligament, with attachment lamella well developed along 2/3 of lateral edge; with wide connecting plates slightly developed; MIII completely pigmented, with attachment lamella well developed along entire lateral edge; MIV completely pigmented, with attachment lamella well developed; MV free, reduced just to attachment lamella, lateral to MIV and MIII. Mandible fused up to 3/4 of its length. Parapodia with dorsal cirrus slightly developed; without branchiae. Chaetae limbate capillaries and simple and compound multidentate hooded hooks. Pygidium with anal cirri.

##### Remarks.

*Lumbrineris* is the most species rich of lumbrinerid genera. It has a long and confused taxonomic history, summarised in the most recent review carried out by Carrera-Parra (2006b), who also provided an updated and restricted definition of this genus, recognising only ~ 35 species as valid. However, it is important to state that according to G. Read (Read in fide 2019 in Read and Fauchald 2022b), many studies, including that of Carrera-Parra (2006b), have cited incorrect type species for this genus; the correct type species is *Nereisebranchiata* Pallas, 1788 now accepted as *Lumbrinerisebranchiata* (Pallas, 1788), a species that Carrera-Parra (2006b) did not recognise as *Lumbrineris*. Solving such taxonomic difficulties is beyond the scope of this study and for practical reasons, we follow the definition as given by Carrera-Parra (2006b). One species consistent with the genus *Lumbrineris* has been found in our CCZ samples.

#### ﻿ Lumbrineris

Taxon classificationAnimaliaEunicidaLumbrineridae

sp. NHM_1741

EC31AB37-B788-546E-9F5A-2B14AABC42F1

Fig. 13A–G

##### Material examined.

NHM_0125, coll. 11 Oct. 2013, AB01, UK-1, EBS, 13.75833, -116.69852, 4080 m, https://data.nhm.ac.uk/object/3c704b88-d8ae-42cb-aeae-73e7a20a70b7; NHM_0129, coll. 11 Oct. 2013, AB01, UK-1, EBS, 13.75833, -116.69852, 4080 m, https://data.nhm.ac.uk/object/067a1dee-adcb-4c7f-8445-b68176b5c41b; NHM_8899, NHM ANEA 2022.851, coll. 2 Nov. 2020, DG05a, NORI-D, Box core, 10.97448, -116.35427, 4260 m, https://data.nhm.ac.uk/object/462dea9c-52cb-48bb-8e60-43c5f4f5d472; NHM_0229, NHM ANEA 2022.818, coll. 15 Oct. 2013, AB01, UK-1, ROV, 13.96467, -116.54988, 4072 m, https://data.nhm.ac.uk/object/9474abbe-8d3a-4db8-9897-ff206977918f; NHM_0972, NHM ANEA 2022.820, coll. 23 Feb. 2015, AB02, UK-1, EBS, 12.57133, -116.6105, 4198 m, https://data.nhm.ac.uk/object/c570a340-f2e6-405d-b0b8-f335c9056fbd; NHM_1308, NHM ANEA 2022.830, coll. 1 Mar. 2015, AB02, OMS, EBS, 12.25733, -117.30217, 4302 m, https://data.nhm.ac.uk/object/2d378a92-05c4-417a-9cc7-1c392baf0db7; NHM_1741, NHM ANEA 2022.819, coll. 11 Mar. 2015, AB02, OMS, EBS, 12.17383, -117.19283, 4045 m, https://data.nhm.ac.uk/object/86010404-eae6-4c58-943a-1761c81fa201; NHM_1896, NHM ANEA 2022.829, coll. 13 Mar. 2015, AB02, OMS, EBS, 12.0415, -117.21717, 4094 m, https://data.nhm.ac.uk/object/acf2aa8f-3ca5-4728-ab42-782576ab57fd; NHM_2318, NHM ANEA 2022.821, coll. 26 Feb. 2015, AB02, OMS, EBS, 12.1155, -117.1645, 4100 m, https://data.nhm.ac.uk/object/83e28d46-6d21-4fe6-a9a6-2c7360cfbaa4; NHM_2374, NHM ANEA 2022.822, coll. 20 Feb. 2015, AB02, UK-1, EBS, 12.51317, -116.60417, 4425 m, https://data.nhm.ac.uk/object/f1cda422-c3a0-47cb-b63a-40a1d92232d7; NHM_3133, NHM ANEA 2022.828, coll. 17 Feb. 2015, AB02, UK-1, EBS, 12.38624, -116.54867, 4202 m, https://data.nhm.ac.uk/object/0f6f9455-d82a-4afa-9a12-5f6771e66763; NHM_3591, NHM ANEA 2022.824, coll. 3 Mar. 2020, RC01, OMS, Box core, 14.06995, -116.57628, 4120 m, https://data.nhm.ac.uk/object/23c92bc4-3099-45b9-bc63-455e094fd5c7; NHM_4237, NHM ANEA 2022.823, coll. 11 Mar. 2020, RC01, UK-1, Box core, 12.37909, -116.55767, 4196 m, https://data.nhm.ac.uk/object/be32a0a9-56ef-41fc-ae65-cf939128372b; NHM_4738_ECDS2, NHM ANEA 2022.825, coll. 28 Feb. 2020, RC01, UK-1, Box core, 13.98698, -116.47664, 4059 m, https://data.nhm.ac.uk/object/cd4b907b-383e-4a71-8ebb-573a8745a8bc; NHM_7057_HW01, NHM ANEA 2022.827, coll. 13 May 2021, DG05d, NORI-D, Box core, 10.38235, -117.12702, 4309 m, https://data.nhm.ac.uk/object/50895550-0014-46d9-b0ba-76b9e392281b; NHM_7249_HW01, NHM ANEA 2022.831, coll. 14 May 2021, DG05d, NORI-D, Box core, 10.37727, -117.15581, 4302 m, https://data.nhm.ac.uk/object/73e121b5-97fb-4d6e-905f-4387236b9cf6; NHM_8796_HW01, NHM ANEA 2022.826, coll. 6 Nov. 2020, DG05a, NORI-D, Box core, 10.32294, -117.18734, 4282 m, https://data.nhm.ac.uk/object/30460632-eb9b-4655-81c2-c1ce3b2cebdd.

##### Description.

**Figure 13.**
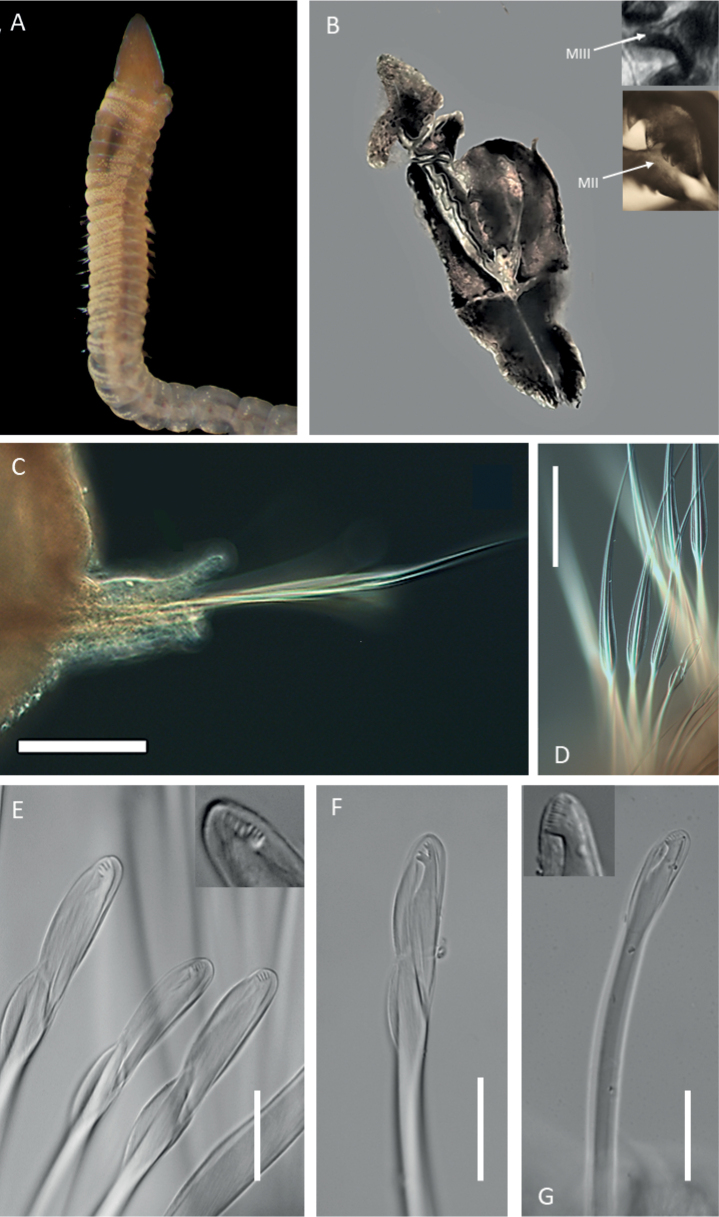
*Lumbrineris* sp. NHM_1741, specimen NHM_1741, unless stated otherwise **A** live anterior fragment of specimen in lateral view **B** maxillary apparatus specimen NHM_0229; insert – the detail of teeth on MII and detail of MIII, showing arcuate edge **C** 22^nd^ parapodium with well-developed postchaetal lobe **D** limbate capillaries on chaetigers 4 and 5 **E** compound hooks on chaetiger 5, insert – detail of the hook dentition **F** compound hook from chaetiger 10 **G** simple multidentate hooded hook chaetiger 12, inset – detail of the hook dentition. Scale bars: 100 µm (**C, D**); 25 µm (**E, F, G**).

Species represented by several posteriorly incomplete specimens. Voucher specimen NHM_1741 (Fig. 13A), 10 mm long 0.8 mm wide for ~ 40 chaetigers long anterior fragment. Live specimens with distinctive spotted pattern across the width of the chaetigers and on prostomium on the dorsum (Fig. 13A). When preserved in ethanol the body is a milky white, retaining iridescent sheen. Body thick in anterior, tapering towards posterior.

Prostomium conical (e.g., specimen NHM_0229) to slightly longer and distally pointed (e.g., specimen NHM_1741) (Fig. 13A), as long or slightly longer than wide; some specimens with prostomium appearing distally truncated (e.g., specimen NHM_2374).

Maxillary apparatus with five pairs of maxillae pigmented pale to dark brown (Fig. 13B). All maxillae with attachment lamellae Carriers ca. equal length as MI and joined entirely to base of MI. MI appears paler with a dark edge; forceps-like, without inner accessory teeth and strongly falcate. MI longer than MII. Connecting plates between MI and MII slightly visible. MII with four pointed teeth, with ligaments (Fig. 13B insert). MIII unidentate, arcuate (Fig. 13B insert), with darkened area on anterior lateral edge. MIV oval shaped and pigmented light brown. MV circular in shape and positioned laterally to MIV. Mandibles fused ~ ½ their length.

Parapodia small and uniramous, increasing in size after the first two chaetigers. Postchaetal lobe longer than pre-chaetal lobe, postchaetal lobe elongated and digitiform, particularly in posterior chaetigers (Fig. 13C). Chaetigers narrow and ring-like until chaetiger 22 where they become longer and more bead-like.

Chaetae characterised by limbate capillaries, simple multidentate hooded hooks and compound multidentate hooded hooks (Fig. 13D–G). Approximately four limbate chaetae in anterior chaetigers (Fig. 13D), increasing in length towards posterior end but decreasing in number. Transition from compound to simple hooks variable (likely size-dependent), with compound multidentate hooded hooks present from chaetiger 1 to 6–15; two or three compound multidentate hooded hooks present. Compound hooks with short blades, with ~ 10 small teeth in lateral view (Fig. 13E) or fewer (Fig. 13F). Simple multidentate hooded hooks appear from chaetigers 8–16, simple hooks only from chaetiger 10 onwards, ~ 2 per parapodia. Simple hooks with ~ 12 small teeth in lateral view (Fig. 13G). Aciculae yellow. Posterior end and pygidium unknown.

##### Genetic data.

This species falls within a low-support clade containing *Lumbrineris* and *Helmutneris* species in our analysis (Fig. 3). There were COI matches on GenBank to four unclassified annelid specimens also collected at the CCZ, GenBank accession numbers KJ736511.1–KJ736514.1 (Janssen et al. 2015).

##### Remarks.

The maxillary apparatus and chaetae composition of this species are indicative of the genus *Lumbrineris* de Blainville, 1828. The strongly arcuate cutting edges of MIII (Fig. 13B) are a typical character of *Lumbrineriscingulata* Ehlers, 1897 (Orensanz 1990; Oug pers. comm., 2023), known from the shelf depths of Magellanic biogeographic province (Carrera-Parra 2006b). Several species reported by Carrera-Parra (2006b), all known from shallow depths, also share this character. CCZ specimens have four teeth on MII, a character further shared by only three known species with MIII with arcuate edge: *L.cingulata*, *L.inhacea* Hartman, 1974 and *L.mustaquimi* Carrera-Parra, 2006b. Some distinguishing characters, mostly based on observation of the hooks, have been summarised in Table 3. CCZ specimens likely belong to a new species that cannot be currently fully formalised; therefore, we assign CCZ specimens to morphospecies *Lumbrineris* sp. NHM_1741.

**Table 3.** Comparison of characters of *Lumbrineris* species with unidentate arcuate MIII and four teeth on MII.

Taxon	Simple hooks from chaetiger/s	Teeth on MII	No. of teeth in composite hooks	No. of teeth in simple hooks	Dorsal cirri	Other characters
*Lumbrineris* sp. NHM_1741	7–16	4 sharp teeth	up to 10	>10	not confirmed?	–
*L.cingulata*	10–20	4 blunt teeth	up to 9	up to 6	present	simple hooks of two sizes, preacicular one bigger
*L.inhacea*	16–20	4 blunt teeth	up to 5	up to 7	present	simple hooks with proximal tooth separated from the others
*L.mustaquimi*	14	4 blunt teeth	up to 10	up to 8	present	first 11 parapodia barely visible dorsally

##### Distribution.

Central Pacific Ocean, Eastern CCZ, found in ‘UK-1’, ‘OMS’ and ‘NORI-D’ exploratory areas (Fig. 1).

## ﻿Discussion

This study presents the morphological and genetic data of six lumbrinerid species from 60 records collected in the eastern CCZ (Table 1). This increases the total number of published annelid species from the targeted areas within CCZ (Fig. 1) to 60, with 18 of them formalised as new species (see Wiklund et al. 2019; Drennan et al. 2021; Neal et al. 2022a, b). Some lumbrinerid species are potentially new to science, but due to suboptimal preservation of the specimens, we were unable to provide formal descriptions of new species. In other cases, the problematic generic definitions prevented formalising new species. Nevertheless, our contribution of genetic and morphological data provides important information, linked to voucher specimens, for future taxonomic studies and surveys in the CCZ.

Genetic data is becoming increasingly important for taxonomic studies, particularly for marine invertebrates which are notoriously difficult to delineate due to high levels of phenotypic plasticity, cryptic species, and morphological stasis (Fontaneto et al. 2015). This is common in annelids (Fontaneto et al. 2015; Cerca et al. 2020; Teixeira et al. 2021), with further issues caused by incomplete and/or damaged specimens, and incomplete sampling from hard to access deep-sea benthic habitats (Fontaneto et al. 2015). With the help of molecular methods, we were able to recover species based on genetic data from damaged specimens, sometimes just body fragments, e.g., Lumbrineridae sp. (NHM_2146) which was unidentifiable by morphology alone.

Annelids are an abundant and important ecological group; therefore, they can be useful in biogeographical studies of the deep sea (Rex and Etter 2010). Two of our CCZ species, Lumbrineridescf.laubieri (NHM_0020) and *Augeneria* sp. NHM_4590, matched species described in the literature based on morphology, with genetic data from type localities currently unavailable. We also recovered matches with some unnamed specimens collected during previous expeditions to the CCZ that had not been assigned a taxon beyond “Polychaeta” (e.g., Janssen et al. 2015). *Lumbrinerideslaubieri* is a deep-sea Atlantic species that may potentially have a distribution in the abyssal Pacific as shown for other CCZ annelid species (e.g., Guggolz et al. 2020; Neal et al. 2022a, b). Further, specimens of Lumbrineridescf.laubieri (NHM_0020) were very abundant in CCZ samples, making them a candidate for future ecological, biogeographical and population genetics studies (see Stewart et al. 2023). On the other hand, *Augeneriabidens* to which CCZ species *Augeneria* sp. NHM_4590 shows similarities, has been originally described from much shallower Atlantic depths (348–642 m), which suggests that CCZ specimens belong to a different species as genetic exchange across ~4000 m depth is unlikely (see Taylor and Roterman 2017 for general discussion), but further taxonomic work is needed. The other four morphospecies included in this study are currently restricted to the CCZ, although future sampling and sequencing may challenge such restricted distribution.

Molecular data for Lumbrineridae are still rare, but recent efforts led to the first published molecular phylogeny for this group (Borisova and Budaeva 2022). By adding genes from six abyssal species included in this study, our molecular phylogenetic analysis recovered several genera as monophyletic - *Augeneria*, *Gallardoneris*, *Ninoe* and *Lumbrinerides* (Fig. 3), as did the previous study (Borisova and Budaeva 2022). In accordance with the published data, *Lumbrineris*, a type genus of Lumbrineridae was shown to be polyphyletic. The taxonomic status of type taxon of *Lumbrineris* is also problematic (see Remarks section of *Lumbrineris* above). The suggested “correct” type species is *Nereisebranchiata* Pallas, 1788, designated by Pettibone (1963) and now accepted as *Lumbrinerisebranchiata.* Genetic data from *L.ebranchiata* are currently unavailable to the best of our knowledge.

We present morphological and molecular data for six abyssal lumbrinerid species, as taxonomic knowledge is paramount to establishing a conservation plan for the deep sea by providing a knowledge of what lives there. Species-level information is critical to robust characterisation of these environments, and building checklists, to allow iterative improvement of this little-known environment (Rabone et al. 2023b). Future studies will be able to use data presented here to build a broader picture of deep-sea biodiversity within the CCZ.

## Supplementary Material

XML Treatment for Lumbrineridae

XML Treatment for Lumbrineridae

XML Treatment for Augeneria

XML Treatment for Augeneria

XML Treatment for Augeneria

XML Treatment for Lumbrinerides

XML Treatment for Lumbrinerides cf. laubieri

XML Treatment for Lumbrineris

XML Treatment for Lumbrineris

## ﻿Additional information

### Author ORCIDs

Lenka Neal https://orcid.org/0000-0002-3857-8428

Helena Wiklund https://orcid.org/0000-0002-8252-3504

Muriel Rabone https://orcid.org/0000-0002-8351-2313

Guadalupe Bribiesca-Contreras https://orcid.org/0000-0001-8163-8724

Eva C. D. Stewart https://orcid.org/0000-0001-8383-5705

Thomas G. Dahlgren https://orcid.org/0000-0001-6854-2031

Adrian G. Glover https://orcid.org/0000-0002-9489-074X

### Data availability

All of the data that support the findings of this study are available in the main text or Supplementary Information.

## Supplementary materials

Supplementary material 1 GenBank sequences Data type table (excel document) https://binary.pensoft.net/file/883776This dataset is made available under the Open Database License (http://opendatacommons.org/licenses/odbl/1.0/). The Open Database License (ODbL) is a license agreement intended to allow users to freely share, modify, and use this Dataset while maintaining this same freedom for others, provided that the original source and author(s) are credited.Lenka Neal, Emily Abrahams, Helena Wiklund, Muriel Rabone, Guadalupe Bribiesca-Contreras, Eva C. D. Stewart, Thomas G. Dahlgren, Adrian Glover

Supplementary material 2 DarwinCore database containing taxonomic designation and collection details Data type table (excel document) https://binary.pensoft.net/file/883777This dataset is made available under the Open Database License (http://opendatacommons.org/licenses/odbl/1.0/). The Open Database License (ODbL) is a license agreement intended to allow users to freely share, modify, and use this Dataset while maintaining this same freedom for others, provided that the original source and author(s) are credited.Lenka Neal, Emily Abrahams, Helena Wiklund, Muriel Rabone, Guadalupe Bribiesca-Contreras, Eva C. D. Stewart, Thomas G. Dahlgren, Adrian Glover
